# Eco-friendly synthesis of Ag/CeO_2_ and CuO/CeO_2_ nanocomposites using *Curcuma longa* extract and assessment of their antioxidant, antifungal, and cytotoxic activities†

**DOI:** 10.1039/d5ra00739a

**Published:** 2025-04-17

**Authors:** Khaled M. Elattar, Abeer A. Ghoniem, Fatimah O. Al-Otibi, Abdulaziz S. Fakhouri, Yosra A. Helmy, WesamEldin I. A. Saber, Mahmoud A. E. Hassan, Ashraf Elsayed

**Affiliations:** a Unit of Genetic Engineering and Biotechnology, Faculty of Science, Mansoura University El-Gomhoria St. Mansoura 35516 Egypt khaledelattar2@yahoo.com khaledelattar2@mans.edu.eg; b Microbial Activity Unit, Department of Microbiology, Soils, Water and Environment Research Institute, Agricultural Research Center Giza 12619 Egypt abeer.abdelkhalik@yahoo.com wesameldin.saber@arc.sci.eg wiasaber@gmail.com; c Botany and Microbiology Department, Faculty of Science, King Saud University Riyadh 11451 Saudi Arabia falotibi@ksu.edu.sa; d Center of Excellence in Biotechnology Research, King Saud University Riyadh 11451 Saudi Arabia; e Department of Biomedical Technology, College of Applied Medical Sciences, King Saud University Riyadh 12372 Saudi Arabia afakhouri@ksu.edu.sa; f Department of Veterinary Science, College of Agriculture, Food, and Environment, University of Kentucky Lexington KY 40546 USA yosra.helmy@uky.edu; g Animal Production Research Institute (APRI), Agricultural Research Center Giza 12619 Egypt m.hassan55213@gmail.com; h Botany Department, Faculty of Science, Mansoura University Mansoura 35516 Egypt ashraf-badawy@mans.edu.eg

## Abstract

This work focused on the biosynthesis of Ag/CeO_2_ and CuO/CeO_2_ nanocomposites (NCs) using *Curcuma longa* extract. The nanocomposites were efficiently characterized using different techniques such as FTIR, UV-visible spectroscopy, zeta potential, DLS, TEM, SEM, EDX, and XRD analyses. The *C. longa* extract provided high phenolic and flavonoid contents, while demonstrating strong antioxidant action at IC_50_ = 0.042 mg mL^−1^. In particular, both nanocomposites exhibited privileged antifungal activity against *Macrophomina phaseolina* with superiority to CuO/CeO_2_ (MIC = 29 µg mL^−1^) over Ag/CeO_2_ (MIC = 49 µg mL^−1^). TEM analyses confirmed the adverse effect of nanocomposites on the fungal cell wall. The CuO/CeO_2_ structure led to mitochondrial and cytoplasmic damage in MCF-7 cells (IC_50_ = 0.5071 µg mL^−1^) according to cytotoxicity tests; however, the Ag/CeO_2_ NC resulted in significant nuclear damage and an increased occurrence of autophagy events. The nanocomposites showed cytotoxic properties by causing oxidative stress, leading to damage of the genomic material and defects in cell structure, suggesting potential therapeutic applications.

## Abstract 2

Ag/CeO_2_ and CuO/CeO_2_ nanocomposites were synthesized using *Curcuma longa* extract and showed notable antioxidant, antifungal activity against *M. phaseolina*, and cytotoxicity toward MCF-7 cells.

## Graphical abstract

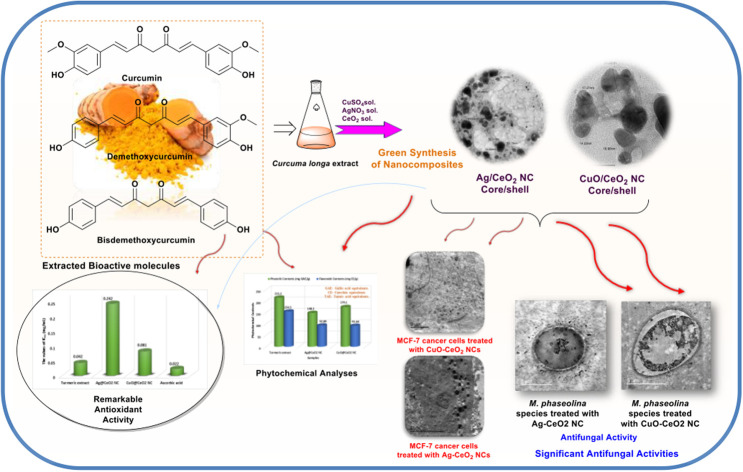

## 1. Introduction

Curcumin, a polyphenol from turmeric (*Curcuma longa* L), has a long history of medicinal use. Traditionally used to treat various ailments, curcumin's primary bioactive component has garnered significant scientific attention.^1^ Research has consistently highlighted curcumin's potential therapeutic benefits in neurological-, respiratory-, cardiovascular-, metabolic-, and autoimmune disorders. Its anti-oxidant, anti-inflammatory, and anti-cancer behavior makes it a promising candidate for applications in these areas. This review will delve deeper into the scientific evidence supporting curcumin's therapeutic potential and explore its promising role in modern medicine.^2^

Curcumin is a symmetric molecule with a heptadiene sequence and two aromatic rings substituted with hydroxyl and methoxy groups.^3^ To elucidate curcumin's natural function, these functional groups are considered, including the *o*-methoxy phenolic group, α,β-unsaturated β-diketone moiety, and 7-carbon chain.^4^ Curcumin's therapeutic activity arises from its promiscuous interaction with various cellular processes. The proteins of interest are multifunctional, encompassing growth factors, transcription factors, inflammatory mediators, pro- and anti-apoptotic pathways, and enzymes, thereby categorizing curcumin as a pleiotropic molecule in disease contexts.^5^ Additionally, curcumin exhibits antioxidant activity and modulates immunological signaling.^5^ Over the past decades, curcumin has garnered significant attention from scientists, particularly in biology, pharmacology, and nutraceutical science.^6,7^ Notably, its antibacterial properties have also been examined.^8^

It has been established that curcumin from the turmeric plant depends on the processing methods used when being extracted. For example, the American Spice Trade Association and the Food and Drug Administration set standard limits regarding the types and amounts of banned compounds present in commercial turmeric powder.^9,10^ Turmeric powder consists of 60–70% total carbohydrates, 6–13% moisture, 6–8% total protein, 5–10% total over, 3–7% total minerals, 3–7% volatile oil, 2–7% total dietary fiber and 1–9% curcuminoids.^10^ Newer studies have isolated over 235 other constituents of turmeric; besides curcumin, it also contains phenolic compounds and terpenoids.^11^

Biomolecules with self-therapeutic properties have gained increasing attention for nanoparticle synthesis because of their biocompatibility, biodegradability, repeatability, and non-toxic nature.^12–14^ These characteristics include the individual surface area of nanoparticles and raises the reactivity of the nanoparticles.^15,16^ However, most conventional methods of synthesizing nanoparticles involve some drawbacks such as high energy depletion, the toxicity of the chemicals used, and the need for high-tech equipment. Hence, more stress has been attached to green synthesis since it is a relatively environmentally friendly synthesis technique.^17,18^

Nanotechnology is a revolutionary technology with the possibility of affecting most sectors. It provides new approaches for controlling biotic and abiotic stresses at the molecular level.^19^ The opportunity to create engineered nanoparticles facilitates new strategies in plant disease control.^20^ The application of nanoparticles mentioned in several studies would enhance the effectiveness of crop protection against phytopathogens.^21^

CuO nanoparticles (NPs) have fair antibacterial efficacy against *Pseudomonas aeruginosa* and *Aeromonas hydrophila*.^22^ These CuO NPs also interact with cancer cells.^22^ Based on this CuO NPs were synthesized and characterized using *Curcuma* extract.^12^ Porous curcumin-copper nanoparticles (Cur/Cu NPs) show antifungal proprieties against *Fusarium oxysporum f.sp. ciceri*. Subsequent studies^23^ on copper-containing nanoparticles exhibited pro-active broad-spectrum antimicrobial properties, along with anti-inflammatory and cytotoxic effects. Other nanoparticle materials in the current research include the utility of nanoparticles for wound healing and anticancer activities.^24^ Another study shows the efficacy of Cuf-TMB@PDA nanoparticles in wound healing.^25^ Recently, the action of nanoceria-curcumin against cancer cells has been demonstrated.^26^ In addition the green synthesis of Ag NPs using Curcuma extract was characterized.^27^

Numerous studies extend the initial findings involving metal oxide nanoparticle anticancer and antimicrobial properties while examining synthesis methods, characterization techniques, and potential therapeutic applications. Metal oxide nanoparticles exhibit potential anticancer characteristics against hepatocellular carcinoma HepG2, which confirms our findings regarding Ag/CeO_2_ and CuO/CeO_2_ nanocomposite cytotoxicity.^28^ The antibacterial and anticancer activities of AgAu bimetal-doped CeO_2_ nanoparticles originate from their ionic-liquid-functionalized biogenic synthesis.^29^ The green synthesized CeO_2_–CuO nanocomposites have anti-cancer properties against Saos-2 osteosarcoma cells,^30^ which is similar to our CuO/CeO_2_ NC research. Cerium oxide particles were used to develop combined antibacterial and anticancer nanotherapeutic systems, which strengthens the understanding of metal oxide nanoparticle antimicrobial and anticancer properties.^31^ Meanwhile, research on metal-based nanoparticles through biological synthesis shows anticancer activities for hepatocellular carcinoma cells.^32^ Green synthesis of cerium oxide nanoparticles shows therapeutic effectiveness and further provides mechanistic anticancer actions.^33^

Building upon these earlier studies, this work focuses on synthesizing Ag/CeO_2_ and CuO/CeO_2_ nanocomposites (NCs) through a green technique involving the utility of *Curcuma longa* extract as a reducing-, stabilizing-, and capping agent. The generated nanocomposites were characterized by UV-Vis, FTIR, XRD, SEM, TEM, zeta potential, and EDX. Additionally, the biological functions of the nanocomposites were investigated, including antioxidant activity, anti-pathogenicity against *Macrophomina phaseolina*, and cytotoxicity against the breast cancer cell line. The use of Ag combined with CuO and CeO_2_ in nanocomposite structure demonstrates novelty because the compounds provide separate yet synergistic therapeutic characteristics. The antimicrobial properties of Ag, along with the cytotoxic effects of CuO, are matched with CeO_2_'s antioxidant character and anticancer functions. Through metal oxide synergies, this nanocomposite combination demonstrates better therapeutic potential, which promotes its use for cancer therapy along with infection control applications. Through this approach, nanomaterial production benefits from sustainable methods that no longer require harmful chemicals and excessive energy usage.

In addition, certain disadvantages need to be acknowledged regarding *C. longa* extract utilization during nanoparticle synthesis. The extract's chemical composition remains inconsistent due to multiple influencing variables, including extraction practices, environmental components, and plant types. The changing composition of the extract affects the predictable outcomes and the reliable results of nanoparticle synthesis. The scaling up of *C. longa* extract as an eco-friendly and economical method requires solutions because standardizing the extract and maintaining process control during large-scale production will be difficult. The bioactivity potential of *C. longa* extract shows promise, but its effects could prove less predictable than standardized synthetic manufacturing methods. The research limitations can be solved by improving extraction protocols and studying scale-up methods for synthesis production.

## 2. Materials and methods

### 2.1. Instruments and reagents

The nanocomposites underwent analysis using multiple testing methods, including FTIR, UV-Vis spectroscopy, zeta potential analysis, SEM-EDX, TEM, and XRD for their structural assessment and morphological and elemental investigations. Sonication combined with centrifugation served as essential tools for preparing and processing the nanomaterials during the experimental procedure. The detailed instrumental data can be found in the ESI File “Section S1”.†

The study obtained all chemicals and reagents from trusted suppliers, including Sigma Aldrich (USA), Fluka (Romania), Biomedical Inc. (USA), El-Nasr Pharmaceutical Chemicals (Egypt), and PIOCHEM (Egypt). The experiment required silver nitrate together with copper sulfate alongside cerium oxide, Folin-Ciocalteau reagent, gallic acid, DPPH, and additional analytical reagents (Section S2†).

### 2.2. Preparation of turmeric extract

Turmeric (*Curcuma longa*) powder was obtained from a local market in Mansoura city, Egypt. A solution of turmeric powder (10 g) in ethanol (100 mL, 70%) was stirred for 2 h at room temperature. The mixture was soaked overnight at room temperature with occasional stirring. The mixture was then filtered, and the extract was immediately stored in a dark glass vessel under cooling to maintain its quality.^34^ The plant extract was freshly used for the preparation of nanocomposites and other analyses (FTIR and UV-visible spectroscopy) and tests (phytochemical profile, antioxidant, and antimicrobial assessments).

### 2.3. Green synthesis of nanocomposites

The silver nanoparticles were prepared by adding dropwise turmeric extract (50 mL, 13.44 mg mL^−1^) to 50 mL AgNO_3_ (1 mM, prepared in deionized water). The mixture was stirred at 55 °C until the solution color changed to brown. Similarly, copper nanoparticles were prepared by dropwise addition of turmeric extract (50 mL, 13.44 mg mL^−1^) to the 50 mL copper sulfate (1 mM, prepared in deionized water) with stirring at 70 °C until a notable color change was accomplished (≈5 h). A suspension of cerium dioxide was prepared in ethanol (10 mL, 1 mM) and sonicated for 1 h at 60 °C. To prepare Ag/CeO_2_ and CuO/CeO_2_ NCs, the cerium dioxide suspension was gradually added to the formed solutions of silver and copper nanoparticles with continuous stirring at room temperature. The mixtures were stirred for 4 hours under heating at 60 °C followed by sonication at 60 °C for 3 h to enable the formation of core–shell nanoparticles and interaction between nanoparticles. For SEM, EDX, and XRD analysis, the mixtures were centrifuged to collect the solid nanocomposites. The solid precipitates were washed with ethanol and deionized water to remove contaminants. The solid nanomaterials were dried at 60 °C for 24 hours.^35^

### 2.4. Quantification of phytochemical analysis

The total phenolic contents were assessed using the Folin–Ciocalteau assay.^36,37^ For the assay, 100 µL of the solution was combined with 5 mL of dilution Folin–Ciocalteau reagent (1 : 10) along with the addition of 4 mL sodium carbonate solution (7.5%). Distilled water was added to reach 10 mL volume, which was then incubated in dark conditions at 40 °C for 30 minutes. Measurement of the absorbance occurred at 765 nm using a spectrophotometer. The measurement of phenolic content relied on a standard gallic acid curve (0–100 mg L^−1^) from which the samples obtained their values through standard curve interpolation. The analysis showed concentrations of gallic acid equivalents as mg GAE per g DW in the dried weight of the tested samples. The total flavonoid contents were determined using the aluminum chloride assay.^38,39^ A 100 µL sample was taken, followed by the addition of 4 mL distilled water and 0.3 mL of sodium nitrite solution (5%) and left to stand for 5 minutes. Then, 0.3 mL of aluminum chloride solution (10% in ethanol) was added to the solution before incubating it for another 5 minutes. Subsequently, 2 mL of 1 M sodium hydroxide solution was added and thoroughly mixed. The mixture received 10 mL of distilled water before heat incubation at room temperature for 15 minutes. The absorbance of the orange solution was measured at wavelength 510 nm using a spectrophotometer. The flavonoid content in the samples was measured using a standard curve of catechin (0–100 mg L^−1^), which allowed the calculation of results from sample absorbance data *via* the standard curve equations. The results were evaluated through milligrams of catechin equivalents, which were expressed per gram of dried sample (mg QE per g DW). All assays were calculated using standard curves (gallic acid standard curve for the estimation of phenolic contents (*y* = 0.0062×, *R*^2^ = 0.987), and catechin standard curve for the estimation of flavonoid contents (*y* = 0.0028×, *R*^2^ = 0.988)) (Section S3†). The analysis was performed in three replicates for *C. longa* extract and nanocomposites. The values were expressed as the mean value ± standard deviation (mean ± SD).

### 2.5. Antioxidant activity

The antioxidant potential was determined using the DPPH˙ method with standard ascorbic acid.^40^ Dilutions of each of the samples were made by serial dilutions in methanol. An equal volume of each experimental sample to be tested was mixed with 0.135 mM of DPPH˙ solution. Each sample was then incubated for 30 min in the dark at room temperature and the absorbance was read at 517 nm. The remaining DPPH˙ (%) was estimated as follows (eqn (1)):

1 % DPPH˙ remaining = [DPPH˙]*T*/[DPPH˙]*T* = 0 × 100

To draw a calibration curve, the percentage of remaining DPPH˙ was plotted against the sample concentration in mg mL^−1^, and the IC_50_ was estimated. The lower the IC_50_ value to which the reagents were obtained, the higher the antioxidant activity of the investigated sample.^41^ The analysis was performed in three replicates, and the values were expressed as the mean value ± standard deviation (mean ± SD) (Section S4†).

### 2.6. Antifungal activity

#### 2.6.1. Fungal species

A strain of *M*. *phaseolina*, a well-known plant pathogenic fungus, was acquired from the Seed Pathology Research Department at the Plant Pathology Research Institute, Agricultural Research Centre, Giza, Egypt. This fungal strain served as a model microorganism for assessing the antifungal properties of the biosynthesized nanomaterials.

#### 2.6.2. Minimum inhibitory concentration (MIC) determination

The MIC (the lowest nanomaterial concentration completely inhibiting visible growth of the fungus) of the nanocomposites (CuO/CeO_2_ and Ag/CeO_2_) against *M. phaseolina* was determined using a broth microdilution assay.^42^ Briefly, sterile potato dextrose agar (PDA) was prepared in flasks, amended with varying concentrations of nanomaterials, and poured into Petri dishes. After solidification, each plate was inoculated with a 0.5 cm *M. phaseolina* disc. Following incubation at 25 °C, fungal growth was monitored for up to 5 days.

### 2.7. Cytotoxic activity

#### 2.7.1. Materials used in tissue culture

Breast cancer cell line (MCF-7) was obtained from NAWAH Company Cairo, Egypt. The cells were cultured in DMEM high glucose (Cat, No. L0103, Biowest, Canada) containing 10% fetal bovine serum (FBS, Cat, No. 10270-098; Gibco, Thermo Fisher Scientific, US), and penicillin/streptomycin (100 µg mL^−1^) (Cat, No. 15140–122, Gibco BRL, Thermo Fisher Scientific, Grand Island, NY). Trypsin (Cat. No. 25200056, 0.25% Trypsin–EDTA) and TRIzol reagent (Cat, No. 15596026) were from Thermo Fisher Scientific, USA. Phosphate buffered saline (PBS, Cat, No. L0615-500) was from Biowest, USA. Dimethyl sulfoxide (DMSO) (Cat, No. D2650) and MTT (Cat, No.M5655) were obtained from Sigma-Aldrich (USA and Munich, Germany, respectively).

#### 2.7.2. Characterization of breast cancer stem cells

Ag/CeO_2_ and CuO/CeO_2_ NCs were first dissolved in appropriate solvents. A stock solution of Ag/CeO_2_ and CuO/CeO_2_ NCs (8 mM) was made in dimethyl sulfoxide (DMSO) and stored at −20 °C until needed. The final nanocomposite concentrations were made by diluting the stock solution in a cell culture medium just before testing. Control experiments using DMSO were also conducted to examine the potential cytotoxic activities of the solvent.

#### 2.7.3. Cell line and cell culture

The MCF-7 human breast cancer cell line was cultured in DMEM supplemented with 10% fetal bovine serum and 1% penicillin/streptomycin maintained in a humidified incubator at 37 °C with 5% CO_2_. When the cells reached 70–80% confluence, they were trypsinized, counted using a hemocytometer with trypan blue staining, and resuspended in fresh media. The viable cells were counted under a 10× magnification microscope. The concentration of viable cells was calculated using eqn (2).2
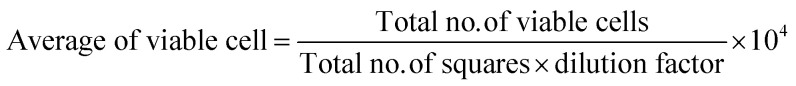


#### 2.7.4. MTT assay

Cell viability was measured utilizing the MTT assay.^43,44^ The cells were seeded in 96-well plates and treated with varying concentrations of Ag/CeO_2_ and CuO/CeO_2_ NCs (8 mM). Following incubation, MTT solution was added, and formazan crystals were formed by viable cells. After dissolving the crystals, absorbance was measured at 570–630 nm using a microplate reader. The IC_50_ values were calculated using GraphPad Prism 8 software. The cell viability was estimated using eqn (3).3
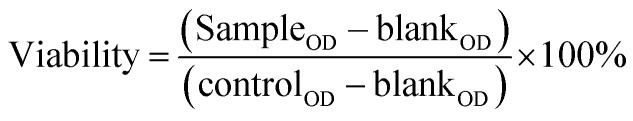


For significant cytotoxicity, a viability threshold of less than 70% was considered, as recommended in previous studies.^45^

### 2.8. Statistical procedure and software

The evaluation of mean data involved the use of Statistical Package for Social Sciences (SPSS, version 21) software. The *p*-value ≤ 0.05 served as the threshold for statistical significance to establish meaningful differences between experimental groups (Section 5).

## 3. Results and discussion

### 3.1. Mechanism of formation of nanocomposites

**Fig. 1:**
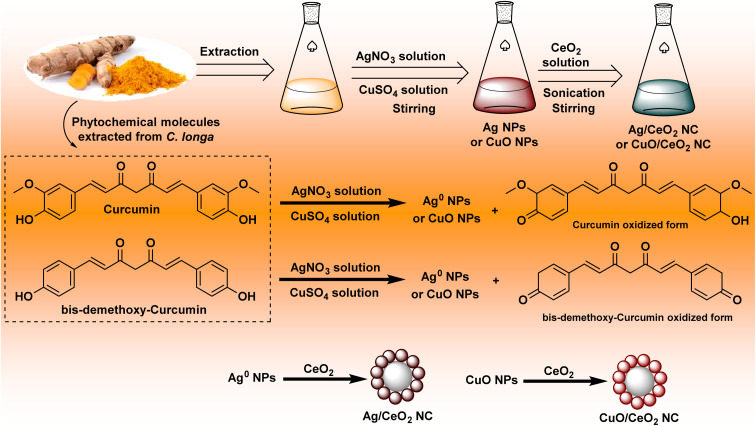
Plausible mechanism for the formation of nanocomposites.

The formation of nanocomposites using *C. longa* extract involved several steps such as reduction and capping by *C. longa* extract, interaction with CeO_2_ NPs, reshaping, and growth of cerium dioxide shell (Fig. 1). The creation of Ag/CeO_2_ and CuO/CeO_2_ NCs has some common features. However, emphasizing their differences offers a better understanding of the entire process of each.

#### 3.1.1 Reduction and stabilization

Curcumin's phenolic hydroxyl groups act as reducing agents for Ag^+^ and Cu^2+^ ions, donating electrons to their respective metallic ions (Ag^0^ and CuO) (eqn (4) and (5)):^46^

4 Ag^+^ + C_14_H_14_O_4_ → Ag^0^ + CO_2_ + H_2_O + other byproducts

5 Cu^2+^ + C_14_H_14_O_4_ → CuO + CO_2_ + H_2_O + other byproducts

Curcumin and other extract components bind to the newly formed metal nanoparticles (Ag NPs and CuO NPs), preventing agglomeration and stabilizing them.^47^ This capping influences their size, shape, and dispersity. The interaction can be described by the Langmuir adsorption isotherm (eqn (6)):

6 *θ* = *K*-ads × *C*/(1 + *K*-ads × *C*)

where *θ* is the surface coverage of metal nanoparticles by curcumin, *C* is the concentration of curcumin in the extract, and *K*-ads is the adsorption constant.

#### 3.1.2 Interaction with CeO_2_ nanoparticles

In the presence of water and the extract, a small fraction of CeO_2_ nanoparticles can dissolve, releasing Ce^4+^ ions into the solution.^48^ These ions can then undergo reprecipitation onto the surfaces of the metal nanoparticles, forming a CeO_2_ shell.

#### 3.1.3 Reshaping and growth of CeO_2_ shell

CeO_2_ nanoparticles that do not dissolve can physically attach to the metal nanoparticles, contributing to the CeO_2_ shell or the particles tend to dissociate and nucleate.^49^ On the other hand, smaller CeO_2_ nanoparticles can dissolve and redeposit onto larger CeO_2_ structures, leading to a more uniform and continuous shell around the metal core.

#### 3.1.4 Partial interdiffusion

Under particular conditions (*e.g*., high temperatures and extended reaction times), some metal ions might partially diffuse into the CeO_2_ lattice, creating a more intertwined core–shell structure.^50^ The initial size and surface area of the CeO_2_ nanoparticles can influence the extent of dissolution and reprecipitation, affecting shell formation. Appropriate mixing can improve the interaction between CeO_2_ nanoparticles and metal nanoparticles, supporting shell formation.

#### 3.1.5 Overall general reactions

Simplified overall reactions for the interactions between metal ions, cerium oxide, and turmeric extract components for the formation of both bimetallic nanoparticles can be described as follows (eqn (7) and (8)):

7 AgNO_3_ + CeO_2_ + C_14_H_14_O_4_ + other extract components → Ag/CeO_2_ NC + CO_2_ + H_2_O + byproducts

8 CuSO_4_ + CeO_2_ + C_14_H_14_O_4_ + other extract components → CuO/CeO_2_ NC + CO_2_ + H_2_O + byproducts + SO_4_^2−^

Curcumin displays slightly higher reducing power for Ag^+^ compared to Cu^2+^, potentially prompting the relative rates of metal ion reduction during the nanoparticle formation.^51^ The surface chemistry and charge distribution of Ag NPs and CuO NPs might vary, leading to variations in their interaction with and deposition on the CeO_2_ clusters.^52^ The different characters between both metals could impact the favorite pathway of formation (physical deposition *vs.* interdiffusion) and the final structure of the nanocomposite.^53^

### 3.2. Characterization of nanocomposites

#### 3.2.1. FTIR spectroscopy

**Fig. 2:**
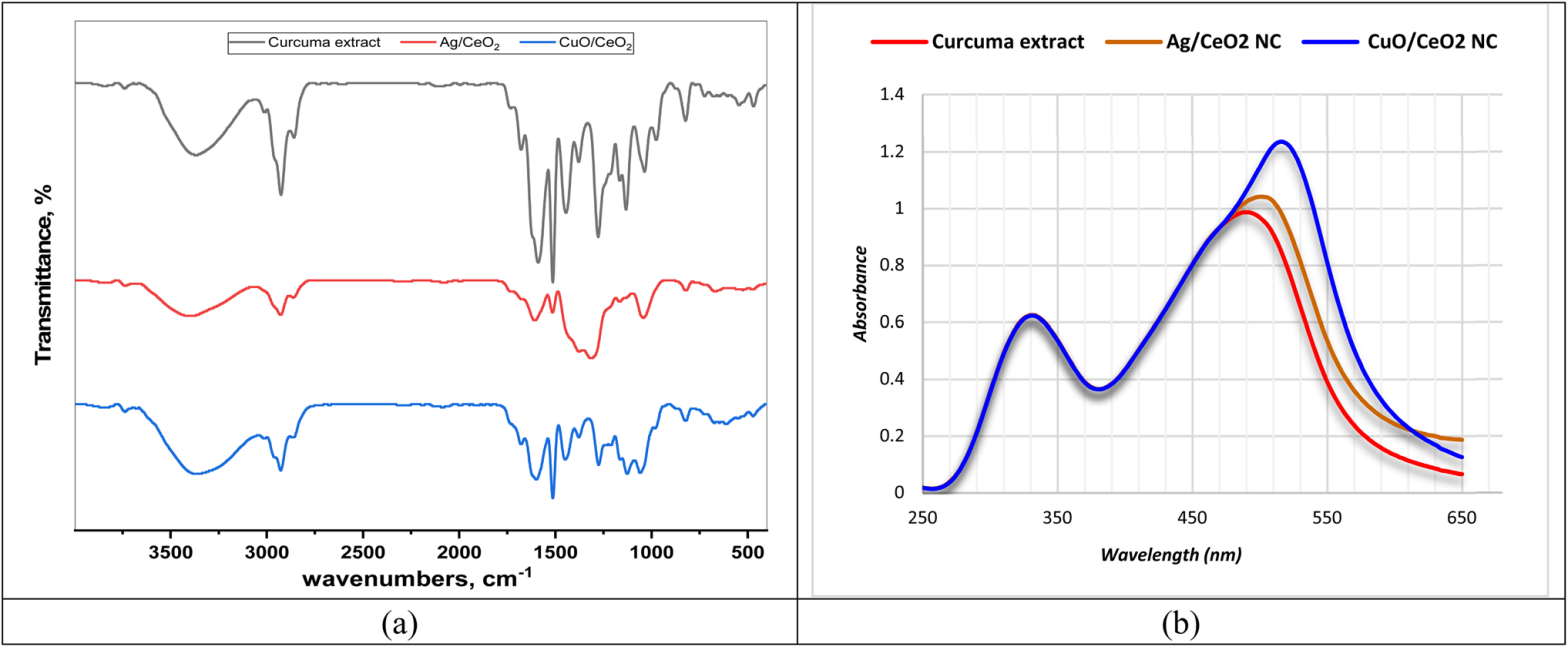
FTIR spectra and UV-visible spectroscopy of the tested samples. (a) FTIR spectra of *C. longa* extract, Ag/CeO_2_, and CuO/CeO_2_ NCs. (b) Absorbance of the samples at different wavelengths.

FTIR spectral analysis was employed to study functional groups of the active phytochemical components in *C. longa* extract that participate in the bioreduction of metal ions to form Ag/CeO_2_ and CuO/CeO_2_ NCs (Fig. 2a and Table S1†). The FTIR spectrum of the *C. longa* extract specified stretching absorption due to hydroxyl groups (O–H) at 3367 cm^−1^ indicated the presence of the hydroxyl group expected from alcohol or phenol compounds. The absorption bands that appeared at 3013, 2965, 2926, 2925, and 2854 cm^−1^ are characterized by the C–H groups, and particularly related to aliphatic groups. Furthermore, a stretching absorption band at 1741 cm^−1^ corresponds to various unsaturated carbonyl (C=O) groups present in curcumin (the primary bioactive component of turmeric) or an alternative component incorporated into ester or carboxyl acid groups. The (C=C) groups of aromatic rings appeared close to 1681, 1626, 1592, and 1572 cm^−1^, implying the presence of aromatic rings, possibly curcumin or alternative aromatic compounds in the mixture, and a conjugated double bond. The absorption band for C–N stretching near 1512 cm^−1^ can be attributed to aromatic amines or nitrogen-rich heterocyclic rings. The range of absorptions between 1455 and 1131 cm^−1^ are attributed to various C–O functional groups, including alcohols, phenols, or esters found in curcumin and other extract components. The fragrance of curcumin and other potential bioactive compounds in the solution remain consistent. The bioreduction of curcumin depends on its hydroxyl-, carbonyl-, and aromatic functional groups which provide two roles: metal ion electron donation and nanoparticle surface stability. The carbonyl groups at 1741 cm^−1^ and aromatic rings at 1681, 1626, 1592, and 1572 cm^−1^ in curcumin powerfully contribute to nanocomposite stability by binding with metal ions and affecting the resulting electronic structure.

The FTIR spectra of Ag/CeO_2_ and CuO/CeO_2_ NCs displayed absorption patterns related to residual organic compounds (C–H stretch) at 2969, 2925, and 2857 cm^−1^ for Ag/CeO_2_, and 3013, 2966, 2925, and 2854 cm^−1^ for CuO/CeO_2_. These absorptions likely originate from stabilizers or adsorbed hydrocarbons used during the synthesis process. The characteristic Ce–O stretching extreme of 1033 cm^−1^ in the FTIR spectrum of Ag/CeO_2_ NCs confirmed the existence of cerium oxide. Like the Ag/CeO_2_ case, the FTIR spectrum of CuO/CeO_2_ NCs showed a combination of functional groups linked at the same time to the metallic oxides (CuO and CeO_2_). The emergence of the Ce–O stretching absorption band indicated the presence of CeO_2_. The presence of the Ce–O stretching band at 1033 cm^−1^ in both Ag/CeO_2_ and CuO/CeO_2_ NCs is a strong indication of the presence of cerium oxide (CeO_2_), as reported in the literature.^54,55^ The FTIR spectra of both types of nanocomposites revealed a complex mixture of functional groups, suggesting the presence of residual organic materials and potential interactions between the metal oxides and organic components. This is consistent with previous studies of Ag/CeO_2_ catalysts prepared using microwave-assisted biosynthesis.^56^ The FTIR spectra from Ag/CeO_2_ and CuO/CeO_2_ nanocomposites show minor spectral changes that indicate identical interactions between their functional groups of carbonyl and aromatic substances. The spectra demonstrate that organic components including curcumin alongside additional phytochemicals stabilize the nanoparticles through their C–O (various) and C–H (organic moieties) active bands.

#### 3.2.2. UV-visible spectroscopy

The UV-visible spectroscopy data offers clues about the potential role of *C. longa* extract in reducing metal ions. *C. longa* extract revealed a peak at 490 nm, likely due to chromophores of curcuminoid compounds. Interestingly, the metal oxide nanocomposites show redshifts compared to the extract. Ag/CeO_2_ NC exhibits a slight red shift of 12 nm, while CuO/CeO_2_ NC shows a larger redshift of 26 nm (Fig. 2b and S1†). These shifts suggest interactions between *C. longa* extract and the nanocomposites formed during bioreduction. Notably, the larger redshift for CuO/CeO_2_ NC hints at a potentially stronger interaction with phytochemical components in the turmeric extract compared to Ag/CeO_2_ NC. This observed interaction aligns with curcumin's known properties. Its antioxidant phenolic hydroxyl groups can donate electrons to metal ions, facilitating their reduction into metal or metal oxide nanoparticles. The extent of the red shift might even be linked to the strength of this interaction between curcumin and the metal ions.

Curcumin's chromophores, responsible for light absorption, can undergo two main types of electronic transitions: n → π* and π → π*. The n → π transition occurs when an electron is excited from a non-bonding orbital (n) to an antibonding pi orbital (π*). This transition typically requires less energy compared to π → π* transitions and often results in weaker light absorption, *i.e*. a lower absorbance value.^57^ During bioreduction, curcumin donates electrons to metal ions, which can modify the energy spectrum levels of the curcumin and metal and move absorption bands toward red wavelengths. The chromophores of curcumin typically undergo n → π* (less energy, weaker absorption) and π → π* transitions (more energy, stronger absorption) in the UV-visible range.^58^ The red shift could be due to phytochemicals donating electrons to metal ions during bioreduction, altering the chromophore's electronic structure and affecting both n → π* and π → π* transitions.^59^

CuO/CeO_2_ NC exhibits a larger redshift than Ag/CeO_2_ NC due to a more robust interaction between curcumin and Cu ions because Cu has a higher electron affinity than Ag. The nanocomposite shows improved interactions between curcumin molecules that would modify their chromophore electronic environment and their absorption behavior. The aggregated state modifies n → π* and π → π* transitions which ultimately results in spectral redshift. The research evidence indicates that *C. longa* extract demonstrates promising potential for biologically reducing metal ions into nanoparticles while the detected spectral redshift explains the electron transfer behavior during metal oxide nanocomposite synthesis.

#### 3.2.3. Zeta potential and DLS analyses

**Fig. 3:**
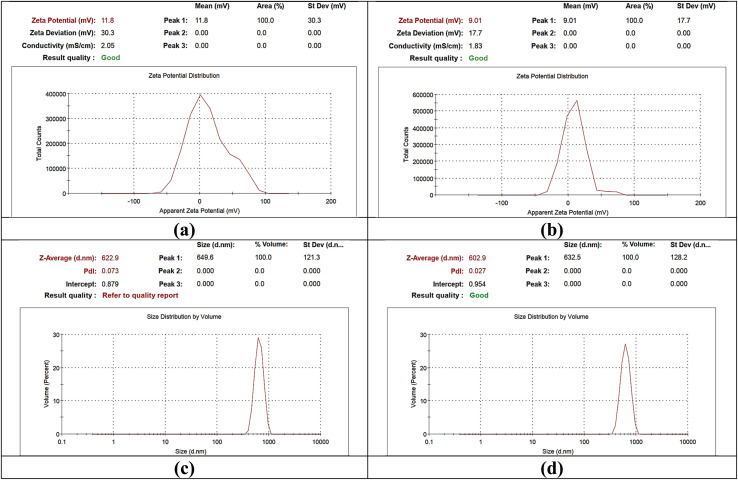
Zeta potential and size distribution of metal oxide nanocrystals. (a) Zeta potential of Ag/CeO_2_ NC; (b) zeta potential of CuO/CeO_2_ NC; (c) size distribution of Ag/CeO_2_ NC; and (d) size distribution of CuO/CeO_2_ NC.

The study of zeta potential and hydrodynamic size dispersion (DLS) provides information on the properties of nanocomposites (Fig. 3 and Table S2†). Together, Ag/CeO_2_ and CuO/CeO_2_ NCs show a slightly constructive zeta capability and propose a net positive charge on their surface. The higher zeta capability of Ag/CeO_2_ NC (11.8 mV compared to 9.01 mV for CuO/CeO_2_) (Fig. 3a and b) suggests strong electrostatic repulsion between the atoms, possibly leading to increased stability. This zeta potential difference can be correlated with the various interactions of curcumin with Ag^+^ and Cu^2+^ ions. Curcumin phenolic groups may bind more strongly with Ag^+^ than Cu^2+^, leading to a higher surface charge and potentially increased stability for Ag/CeO_2_ NCs.

While Ag/CeO_2_ and CuO/CeO_2_ NCs exhibit comparable hydrodynamic sizes (622.9 d nm for Ag/CeO_2_ and 602.9 d nm for CuO/CeO_2_), CuO/CeO_2_ NC exhibit an excessive uniform atom size circulation as demonstrated by a low PDI value (0.027 compared to 0.073 for Ag/CeO_2_) (Fig. 3c and d). The conduction principles of both types of nanoparticles are comparable (2.05 mS cm^−1^ for Ag/CeO_2_ and 1.83 mS cm^−1^ for CuO/CeO_2_) and propose a similar ionic dispersion. Frequently, this difference in zeta potential and atom size may influence the stability and communication of the nanoparticles in different objectives. Our finding indicate a slightly positive zeta probability for the same type of nanocomposites, which is consistent with previous findings for Ag/CeO_2_ NC.^60,61^ The developed positively charged solution has potential applications in dye degradation networks where electrostatic forces enable the breakdown of negatively charged pollutants. We detected strong electrostatic repulsion between Ag/CeO_2_ atoms because Ag/CeO_2_ demonstrates a higher zeta capability than CuO/CeO_2_. The results support the zeta potential theory that elevated zeta potential values provide enhanced colloidal stability, although such nanoparticles would benefit from excellent dispersal properties in applications. Both Ag/CeO_2_ and CuO/CeO_2_ NCs have an equivalent hydrodynamic size of approximately 600 nanometers, which is in line with the expectations of this type of nanomaterial.^62^ The low PDI value (0.027) for CuO/CeO_2_ compared to Ag/CeO_2_ (0.073) indicates an excessive uniform atom size dispersion, and circulation which is desirable for several purposes. The current characteristic may be advantageous for functions where stable atom sizes are essential, such as for targeted drug distribution or catalysis. For functions where stable atom sizes are essential, such as for targeted drug distribution or catalysis, this characteristic may be advantageous. The current analysis suggests that CuO/CeO_2_ NC may have a positive zeta capability.^63^ The present condition is stable with the general shift in the composition of metallic oxide nanoparticles with a constructive veneer charge planned in the presence of a metallic oxide chemical bond. The DLS value of CuO/CeO_2_ NC (602.9 d nm) is comparable with the size demonstrated in previous literature for similar nanomaterials.^60,61^

#### 3.2.4. Transmission electron microscopy (TEM)

**Fig. 4:**
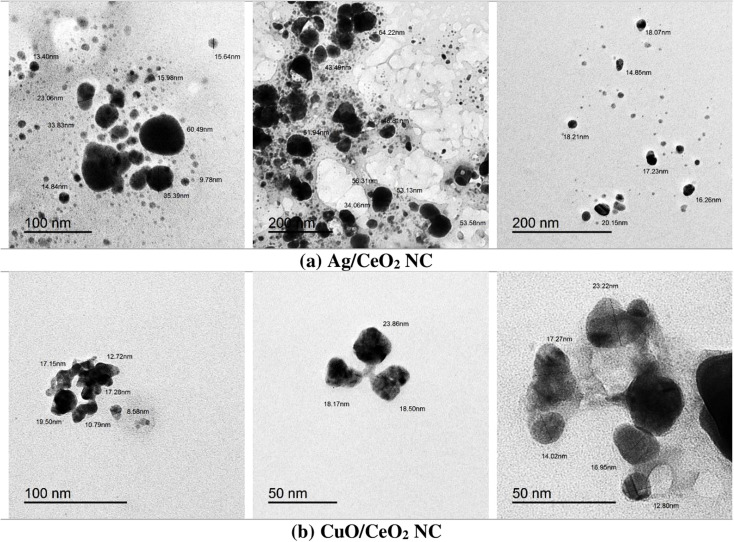
TEM micrographs of (a) Ag/CeO_2_ and (b) CuO/CeO_2_ NCs. Darker regions correspond to silver (Ag) or copper oxide (CuO) nanoparticles, while the lighter matrix represents the CeO_2_ support.

The TEM micrographs of CuO/CeO_2_ NC exhibit dispersion of spherical or slightly elongated nanoparticles with fairly uniform dispersion (Fig. 4a). The original size of such nanoparticles was within the range of approximately 10.79 to 23.86 nm, thus depicting a well-controlled synthesis procedure. Most of the nanoparticles were individualized but a certain amount of aggregation was also observed, which may be attributed to the particle interface characteristics, dispersion medium, and the van der Waals forces. The TEM of Ag/CeO_2_ NC (Fig. 4b) was also observed in a similar size and morphology pattern as the CuO/CeO_2_ NC to infer that the synthesis procedure was analogous. Compared to CuO/CeO_2_ NC, the average size of Ag/CeO_2_ NC in the synthesized composites was slightly greater, varying between 13.4 nm to 64.22 nm. Particle growth analysis showed that the factors controlling particle aggregation were similar in CuO/CeO_2_ and Ag/CeO_2_ NCs. The two clusters of nanocomposites had a spherical or oblong shape with a rather narrow size distribution. The average size of the Ag/CeO_2_ NC was somewhat higher than CuO/CeO_2_ NC. The aggregation occurred in both nanocomposites, implying that the same force affected the particle–particle interactions.^64^ TEM analysis further confirms that both CuO/CeO_2_ and Ag/CeO_2_ NCs were synthesized with the preferable morphology.^65^ The observed aggregation of both nanocomposites can be due to van der Waal forces, electrostatic forces, and surface ligands present in a molecule.

#### 3.2.5. Scanning electron microscopy (SEM)

**Fig. 5:**
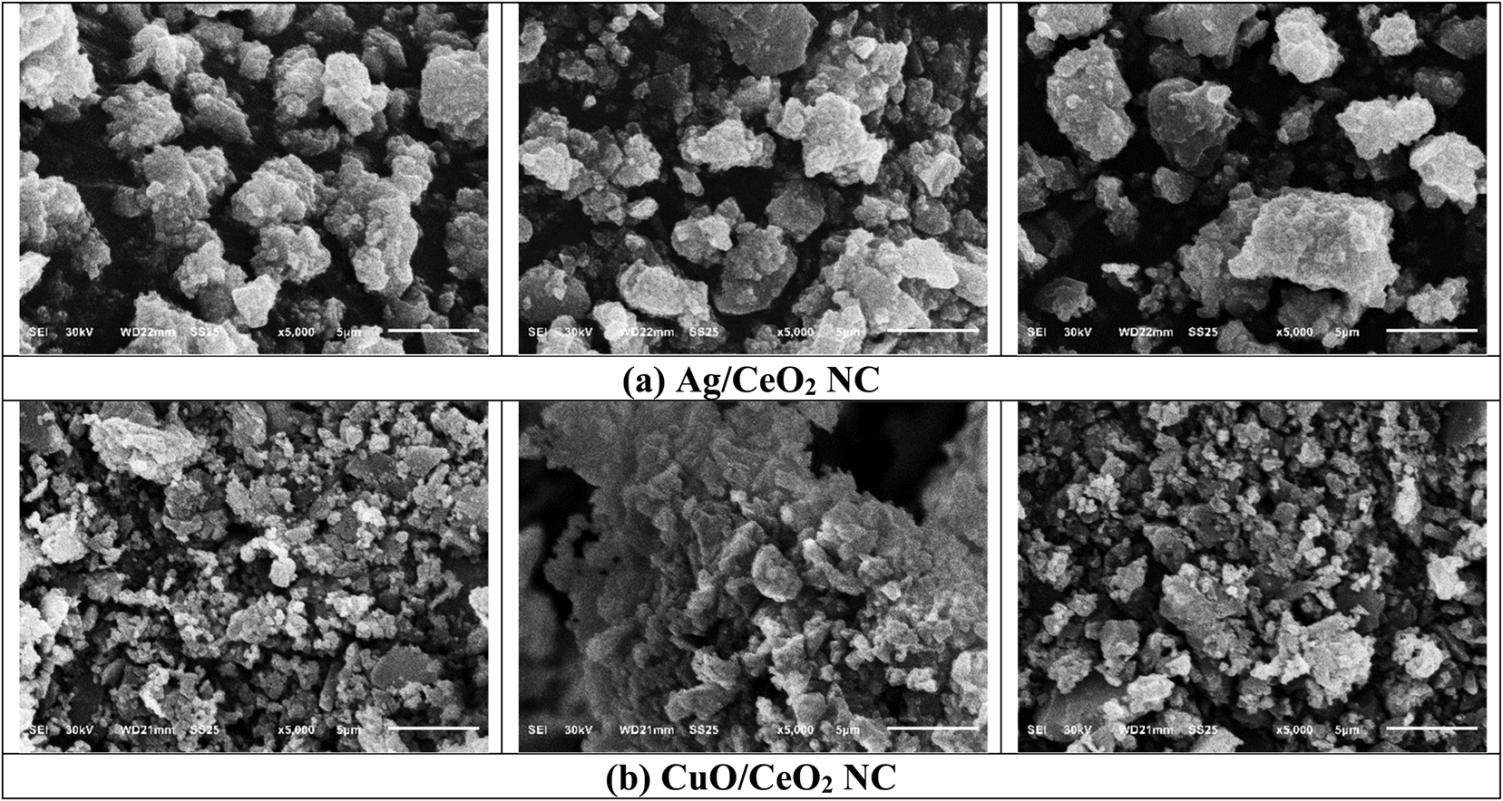
SEM micrographs of (a) Ag/CeO_2_ and (b) CuO/CeO_2_ NCs. All images are scanned with 5000× magnification. Highly dispersed Ag or CuO nanoparticles on the CeO_2_ matrix indicate the successful synthesis of nanocomposites.

The surface structure of Ag/CeO_2_ and CuO/CeO_2_ NCs was investigated using SEM (Fig. 5). Both nanocomposites generally exhibit a spherical or slightly elongated morphology, indicating a successful synthesis process. The nanoparticles in both nanocomposites show a relatively uniform size distribution, with some variation depending on the specific synthesis conditions. Some degree of aggregation is often observed, which can be due to particle surface properties, dispersion conditions, and van der Waals forces. The SEM images may reveal a heterogeneous mixture of particles, suggesting variations in composition or size within the nanocomposites. Both nanocomposites exhibit similar morphologies, suggesting comparable synthesis conditions and particle formation mechanisms. The size distribution of Ag/CeO_2_ NC might differ slightly from that of CuO/CeO_2_ NC, contingent on the specific synthesis limitations and the properties of the metal oxides. The degree of aggregation can vary between the two types of nanocomposites, affected by issues such as particle surface chemistry and the presence of surfactants or capping agents.^66^

#### 3.2.6. Energy-dispersive X-ray spectroscopy (EDX)

**Fig. 6:**
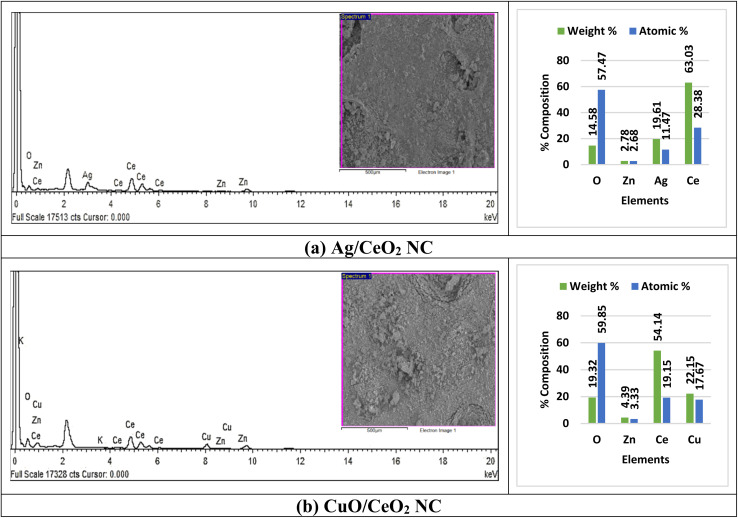
EDX analyses of (a) Ag/CeO_2_ and (b) CuO/CeO_2_ NCs.

The elemental composition in the nanocomposites was explored using EDX analysis to evaluate how the elemental ratios affect the properties of the nanocomposites. Fig. 6 confirmed the successful incorporation of the target elements. In the Ag/CeO_2_ NC, the presence of oxygen, zinc, silver, and cerium showed the following weight (%) and atomic (%): 14.58% and 57.47%, 2.78% and 2.68%, 19.61% and 11.47%, and 63.03% and 28.38%, respectively (Table S3†). Similarly, CuO/CeO_2_ NC had oxygen weight (%) and atomic (%) content of 19.32% and 59.85%, potassium at 0.82% and 1.04%, copper at 22.15% and 17.67%, zinc at 4.39% and 3.33% and cerium at 54.14% and 19.15%, respectively. The minor extent of Zn and K could be attributed to the synthesis conditions or employing specific precursors for the preparation of the nanomaterial.

EDX analysis of the synthesized Ag/CeO_2_ NC showed the presence of oxygen and cerium at higher intensity and silver at moderate intensity while zinc was observed as an impure element at a very low intensity. The EDX analysis of CuO/CeO_2_ NC synthesized using hydrothermal method pointed out that oxygen, copper, and cerium exhibited high-intensity peaks in the X-ray spectrum, whereas potassium was an impurity of low intensity. The EDX data obtained for Ag/CeO_2_ and CuO/CeO_2_ NCs (Fig. 6) aligns well with the findings reported in the literature. Our observation of the dominant oxygen, cerium, and silver is consistent with previous studies on Ag/CeO_2_ NC.^67,68^ The detection of zinc as a minor impurity is in line with the potential for contamination during the synthesis process, as reported in the literature.^69^ The dominance of oxygen, copper, and cerium in the CuO/CeO_2_ NC is expected based on its composition. The presence of potassium as a minor impurity could be attributed to the synthesis conditions or the use of specific precursors.^69^ Briefly, the EDX data obtained in this study is consistent with the literature on Ag/CeO_2_ and CuO/CeO_2_ NCs. The prosperous combination of the desired elements and the identification of minor impurities provide valuable information on the synthesis process and the properties of these materials.

#### 3.2.7. X-ray diffraction (XRD)

**Fig. 7:**
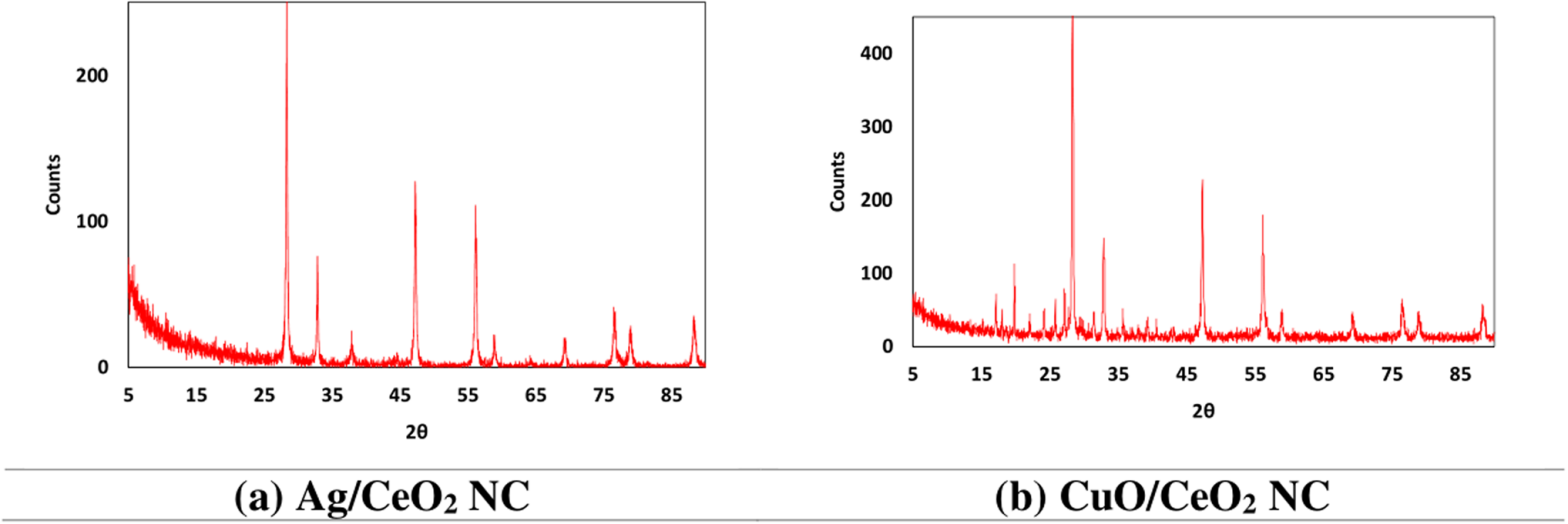
XRD patterns of (a) Ag/CeO_2_ and (b) CuO/CeO_2_ NCs.

The XRD pattern of the Ag/CeO_2_ NC (Fig. 7a and Table S4†) revealed the existence of multiple peaks, confirming that the material is polycrystalline. The first intense peak occurs at the 2*θ* diffraction angle of about 28.34° and its diffraction plane corresponds to a *d*-spacing of about 3.1494 Å. The XRD pattern of the Ag/CeO_2_ NC shows that the sample contains peaks related to (111), (200), (220), (311), (222), (400), (331), (420), (422), and (440) plane of face-centered cubic (fcc) Ag and CeO_2_. Their peak intensities vary, indicating that the crystallites in the nanocomposite have a certain preferred orientation. This has been proved by the fact that the observed *d*-spacing values agree with the standard lattice parameter of fcc Ag and CeO_2_. The observed broadening of the diffraction peaks suggests the nanocrystalline character of the material, with a probable average crystallite size of about 10–20 nm. The XRD result gives credence to the synthesis of Ag/CeO_2_ nanocomposite with a polycrystalline nature, which directs the orientation of the nanocomposite and suggests its suitability for further applications. All the other peaks can be indexed to those of the reference patterns for CeO_2_ (reference code 01-075-0125) except one peak at 37.95°, which supports the identification of CeO_2_ as the main crystalline phase present. The obtained *d*-spacing values that have been calculated are fairly close to the reference pattern for CeO_2_, thus confirming the identification. The selected peak at 28.34° associated with the CeO_2_ (111) plane has the highest value for intensity and relative integrated intensity; therefore, the most preferred crystalline orientation in the sample is the (111) plane.

The XRD pattern of the CuO/CeO_2_ NC has the following characteristic peaks belonging to the face-centered cubic (fcc) CuO and CeO_2_: (111), (200), (220), (311), (222), (400), (331), (420), (422), and (440) (Fig. 7b). The heights of these peaks indicate that there is a favored orientation of the crystallites within the nanocomposite (Table S5†). These values of *d*-spacing are in close agreement with the standard values of fcc copper oxide and cerium oxide, indicating the existence of these phases in the nanocomposite. The characterized broadening of the diffraction peaks gives evidence of the nanocrystalline structure of the synthesized material and the approximate diameter of the crystallites is 10–20 nm. The presence of CeO_2_ was confirmed owing to peaks corresponding to reference patterns (ICSD # 00-050-0543). A few peaks coincide with CuO (ICSD reference code # 01-073-0603), meaning that there is also a copper oxide phase. The determined *d*-spacing values correspond well to the earlier patterns for CeO_2_ and CuO. Based on the XRD results, we can confirm the synthesis of CuO/CeO_2_ NC with a polycrystalline nature. Knowledge of the crystal phases as well as their preferred orientation plays a significant role in defining the structure and possible uses of this nanocomposite.

The XRD data collected for the Ag/CeO_2_ and CuO/CeO_2_ NCs examined in this study are supported by the data available in the literature. This observation of the CeO_2_ phase having major peaks matching (111), (200), (220), and other planes of the crystal face matches previous work.^70^ The appearance of peaks related to Ag at reduced intensity indicates that the Ag has replaced some of the Ce sites in the CeO_2_ perovskite structure as confirmed in another related study.^71^ The values of *d*-spacing patterns estimated for the Ag/CeO_2_ NC correspond with the standard patterns of CeO_2_, indicating that CeO_2_ is the main crystalline phase in the nanocomposite. The XRD diffraction pattern of the CuO/CeO_2_ NC confirms the existence of Cu and Ce phases.^72^ The observed *d*-spacing values also correspond to the standard lattice parameters for the two identified phases, CuO and CeO_2_. The relative intensity of the peak indicates that the preferred orientation of the crystallites in the nanocomposite is identical to Ag/CeO_2_ NC. In general, the results of the XRD analysis observed in this work correlate with the previous reports concerning Ag/CeO_2_ and CuO/CeO_2_ NCs. The material's structural characteristics and potential applications were elucidated by identifying the crystal phases and preferred orientation.

### 3.3. Phytochemical contents

**Fig. 8:**
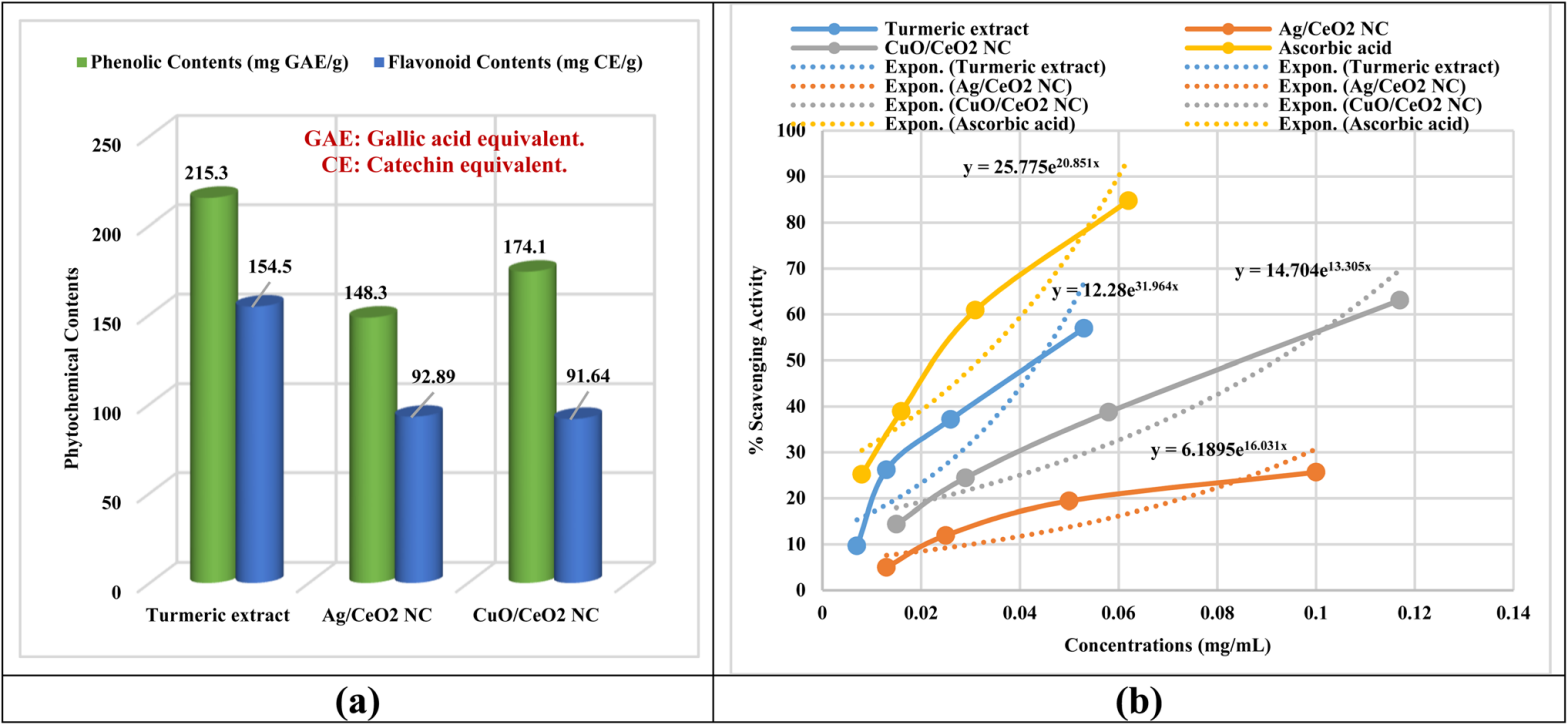
(a) Phytochemical composition of the investigated samples. (b) Antioxidant results.

The phytochemical screening (Fig. 8a and Table S6†) of the extract of turmeric and of Ag/CeO_2_ and CuO/CeO_2_ NCs showed moderate to good content of phenolic and flavonoid compounds. The total flavonoids were estimated to be 155 ± 9.7 mg CE per g and there was 215.3 ± 11.4 mg GAE per g in the turmeric extract. Phenolic content analysis of the NCs showed that Ag/CeO_2_ NC had 91.64 ± 8.46 mg CE per g flavonoids, 148 mg CE per g; the turmeric extract had 92.89 ± 13.40 mg CE per g flavonoids, and the CuO/CeO_2_ NC had 174.1 ± 7.46 mg GAE per g phenolic content. The phytochemicals present in all the nanocomposites and extracts used in this study were presented in appreciated quantity. The slight decrease in phytochemical content of the Ag/CeO_2_ and CuO/CeO_2_ NCs compared to the turmeric extract was most probably due to the contribution of the phytochemicals in the bioreduction process of metal ions.^73^ Phenolic and flavone compounds have high antioxidant activity because of their high electron donation capacities. This characteristic renders the turmeric extract used in this work suitable for the bioreduction process of metal ions to metallic nanoparticles. The bioreduction process results in the transformation of phytochemical components into oxidized or dehydrogenated forms.^74^

In this work, the participation of phenolic compound phytochemicals has been described for the green synthesis of metal nanoparticles. The higher level of phytochemicals in the turmeric extract compared to the nanocomposites can provide evidence that a major part of these compounds was involved in the bioreduction process. The action of phytochemicals with metal ions during the synthesis process may affect the nanoparticle attributes, including size, shape, and stability. This means that although the phytochemical content is lower in the nanocomposites, their bioactivity may be retained, at least in part, by some components or interactions with the nanoparticles or the other phytochemicals.^75^ Therefore, a decline in the phytochemical content of both Ag/CeO_2_ and CuO/CeO_2_ NCs is believed to have been triggered by the bioreduction of metal ions.

### 3.4. Antioxidant activity

The antioxidant activity of turmeric extract and the nanocomposites was assessed by DPPH assays (Fig. 8b). The IC_50_ represents the concentration of the samples that are required to scavenge DPPH radical by 50%. Turmeric extract had the lowest IC_50_ value at a concentration of 0.042 mg mL^−1^ (Fig. S2 and Table S7†), signifying that it was the most active antioxidant among the samples. The IC_50_ of Ag/CeO_2_ NC is even higher (0.242 mg mL^−1^) than the IC_50_ of the turmeric extract, indicating a lower antioxidant capacity. The IC_50_ value for CuO/CeO_2_ NC (0.081 mg mL^−1^) is lower than Ag/CeO_2_ NC but higher than that of turmeric extract. In this case, turmeric extract is the most potent antioxidant agent, followed by CuO/CeO_2_ NC, while Ag/CeO_2_ NC was the least active agent to scavenge the free radicals.

The antioxidant activity of the turmeric extract and nanocomposites are comparable to that of ascorbic acid (IC_50_ = 0.022 mg mL^−1^), confirming their strong antioxidant properties. On the other hand, the highest antioxidant activity was observed with turmeric extract, with a scavenging activity of 56.99% at 0.053 mg mL^−1^. Ag/CeO_2_ NC nanocomposite exhibited significant scavenging activity of 25.67% at 0.1 mg mL^−1^. CuO/CeO_2_ NC also displayed notable scavenging activity of 63.10% at 0.117 mg mL^−1^ (Fig. 8b). All samples exhibited concentration-dependent antioxidant activity, with turmeric extract exhibiting the highest scavenging activity at all concentrations. Both Ag/CeO_2_ and CuO/CeO_2_ NCs displayed significant antioxidant activity, suggesting that the incorporation of the metal oxide nanoparticles did not completely diminish the antioxidant properties of the turmeric extract.

The antioxidant activity of both turmeric extract and the Ag/CeO_2_ and CuO/CeO_2_ NCs can be attributed to their ability to neutralize free radicals, chelate metal ions, and enhance antioxidant activities.^76^ Turmeric extract is rich in phytochemicals such as curcuminoids^77–79^ and directly scavenges free radicals, chelates metal ions, and improves antioxidant activity. The nanocomposites likely retain some phytochemicals from the turmeric extract, although they may also exhibit synergistic effects due to the combination of metal oxides and cerium oxide. Additionally, the surface properties of the nanoparticles and their potential to reduce metal ions could contribute to their antioxidant activity. The results of the antioxidant activity agree with the results of phytochemical contents, indicating the participation of phytochemicals in the bioreduction process to form metal nanoparticles and oxidized or dehydrogenated forms of the phytochemicals of the turmeric extract.

### 3.5. Antifungal activity

#### 3.5.1. Minimum inhibitory concentration (MIC)

Interestingly, the study revealed a greater susceptibility of *M. phaseolina* to both nanocomposites. The MIC values for CuO/CeO_2_ (29 µg mL^−1^) and Ag/CeO_2_ (49 µg mL^−1^) represent the minimum concentrations required to inhibit the growth of *M. phaseolina*. Potential reasons for this difference could be that the cell wall is more susceptible to penetration by the nanoparticles due to its specific structure. *M. phaseolina* might have a higher uptake rate of the nanoparticles, leading to a more significant internal effect. The nanoparticles might interact more effectively with certain cellular processes in *M. phaseolina* that are crucial for its growth and survival. The observation of some fungal growth at lower concentrations suggests a dose-dependent effect of the nanoparticles. Investigating potential synergistic effects between CuO/CeO_2_ and Ag/CeO_2_ NCs with other antifungal treatments could lead to enhanced efficacy and reduced development of resistance.^80,81^

The growth patterns of *M. phaseolina* appear in Fig. S3,† both with and without nanocomposite treatment. *M. phaseolina* maintains its typical network growth pattern before treatment, as an essential reference point to assess antifungal efficacy. The extensive growth shows that no antimicrobial treatment has been applied, which underscores the necessity for antifungal interventions. The application of Ag/CeO_2_ NC to fungal samples caused significant growth reduction while creating visible inhibited areas at their application points. The antimicrobial effect occurs thanks to silver (Ag) nanoparticles interacting with cerium oxide (CeO_2_) to produce reactive oxygen species (ROS) along with supporting redox reactions. Plant protection through sustainable antifungal treatment could be achieved by using Ag/CeO_2_ NC. The antifungal efficiency of CuO/CeO_2_ NC became evident as they created distinct inhibition zones because the ROS that was produced led to fungal cell damage and stopped fungal expansion. The combination of nanoparticles in both materials demonstrates valuable prospects to protect plants from *M. phaseolina* infection.

#### 3.5.2. FTIR spectra of *M. phaseolina* treated with NCs

**Fig. 9:**
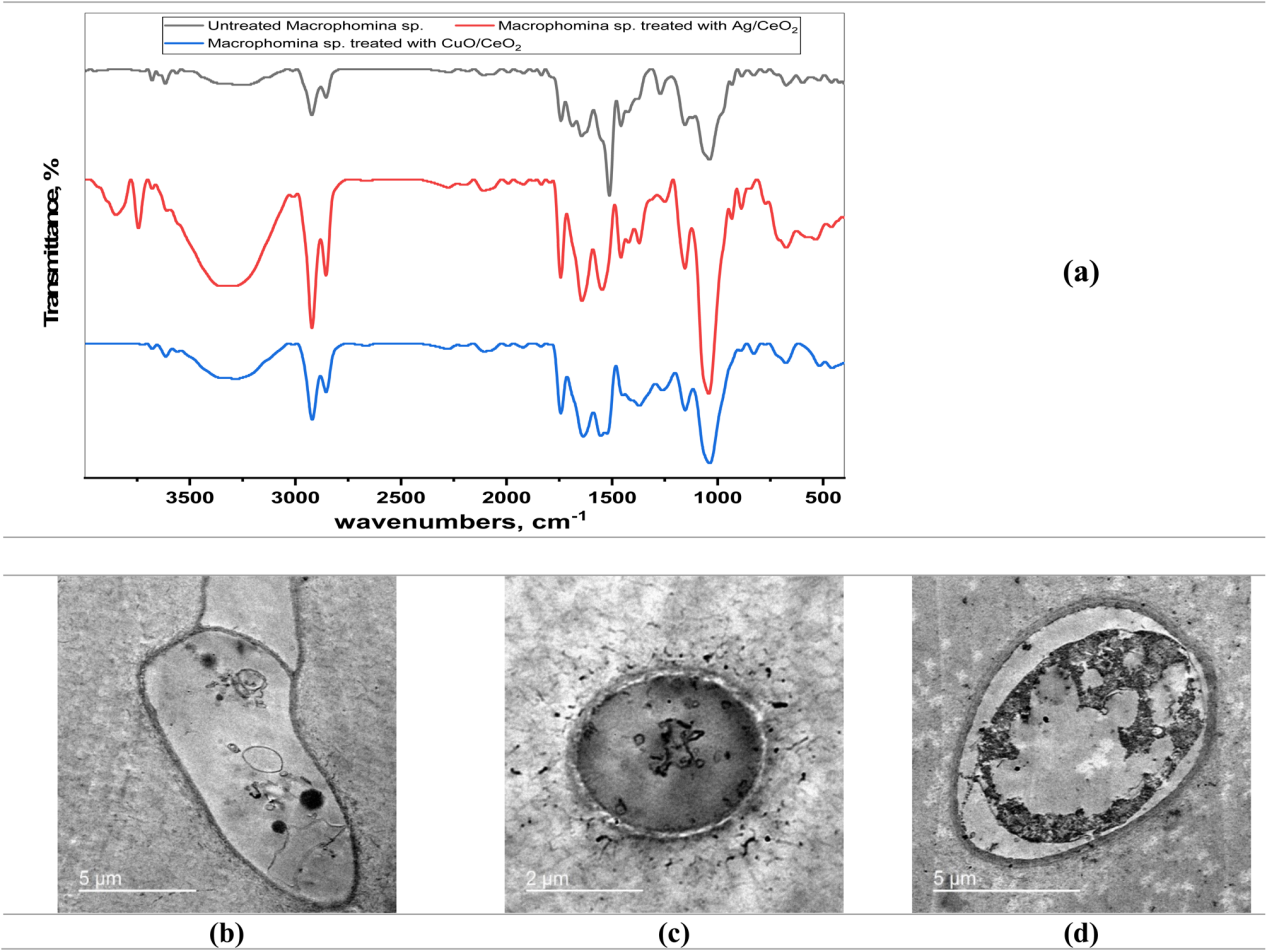
(a) FTIR spectra and (b–d) TEM micrographs of *M. phaseolina* treated and untreated with Ag/CeO_2_ and CuO/CeO_2_ NCs. (b) Untreated *M. phaseolina*. (c) *M. phaseolina* treated with Ag/CeO_2_ NC. (d) *M. phaseolina* treated with CuO/CeO_2_ NC.

The analysis of FTIR was conducted to investigate the potential impact of Ag/CeO_2_ and CuO/CeO_2_ NCs on the chemical composition of *M. phaseolina* (Fig. 9a and Table S8†). The FTIR spectrum of untreated *M. phaseolina* reveals various functional groups, indicating the presence of different biomolecules. The absorption band around 3274 cm^−1^ indicates the presence of O–H stretching, probably from carbohydrates, proteins, and/or water.^82^ The absorption bands between 2923 and 2852 cm^−1^ represent C–H stretching vibrations of aliphatic chains, possibly from fatty acids or lipids in the fungal cell. The absorption bands around 1648 and 1558 cm^−1^ indicate the presence of amide groups, suggesting proteins within the fungal biomass. The FTIR spectra of *M. phaseolina* treated with both Ag/CeO_2_ and CuO/CeO_2_ NCs show some differences compared to the untreated control. The FTIR spectrum of *M. phaseolina* treated with Ag/CeO_2_ shows new absorption bands at 3357 and 1743 cm^−1^, potentially indicating changes in hydrogen bonding or carbonyl groups.^83^ Additionally, the disappearance of some peaks compared to the control suggests potential alterations in specific biomolecules. The FTIR spectrum of *M. phaseolina* treated with CuO/CeO_2_ NC is similar to that of Ag/CeO_2_ NC treatment, and it shows a new absorption band at 1743 cm^−1^ and potentially altered peak intensities compared to the control. The presence of an absorption band at 2663 cm^−1^ might suggest the introduction of new C–H stretching vibrations,^84^ possibly from the nanoparticles themselves. The observed changes in the FTIR spectra (Fig. 9a and Table S8†) suggest that both Ag/CeO_2_ and CuO/CeO_2_ NCs treatments might interact with the chemical composition of *M. phaseolina*. We investigated the TEM micrographs of the *M. phaseolina* fungal species treated and untreated with nanocomposites to explore the mechanism of action for the inhibition of fungal growth.

#### 3.5.3. TEM of *M. phaseolina* treated with NCs

The TEM image of untreated *M. phaseolina* (Fig. 9b) showcases its intricate cellular details. The internal structures like ribosomes and mitochondria are visible within the cytoplasm. TEM analysis reveals potential growth disruption in *M. phaseolina* treated with both Ag/CeO_2_ and CuO/CeO_2_ NCs (Fig. 9c and d). Dark spots are observed on or near the cell wall, likely representing the nanoparticles themselves. Notably, CuO/CeO_2_ treatment exhibits clear deformations in the cell wall, hinting at successful nanoparticle penetration and potential loss of internal turgor pressure, mimicking a plasmolysis-like effect.^85,86^ This internalization is consistent with findings in other studies^24,60^ and could disrupt crucial organelles within the fungus. The observed cell wall damage, potential loss of internal pressure, and disruption of internal structures likely contribute to the inhibited growth of *M. phaseolina*, with CuO/CeO_2_ NC potentially causing more severe damage due to their ability to penetrate the cell wall.

Both nanocomposites exhibit different concentrations of potency because CuO/CeO_2_ shows increased catalytic behavior for reactive oxygen species generation that produces oxidative stress and cell damage. TEM analysis confirms membrane disruption because it shows visible cell wall deformities and both nanoparticle penetration and potential internal turgor pressure loss. The combined findings show that the antifungal effects of CuO/CeO_2_ NCs result from an interaction of oxidative stress and dual damage effects on cell walls and fungal cell structure.^87^ Future experimental research needs to incorporate both ROS measurement tests and membrane stability examinations to prove that oxidative stress exists when validating these results. The integration of nanocomposite antifungal agents with traditional antifungal therapies should be studied because such combinations could improve effectiveness without boosting resistance. Assessing nanocomposites for agricultural sustainability^88^ requires a complete environmental impact evaluation that considers their persistence time along with their toxic effects on beneficial organisms and their ability to biodegrade.

### 3.6. Cytotoxic activity

#### 3.6.1. Cell viability

**Fig. 10:**
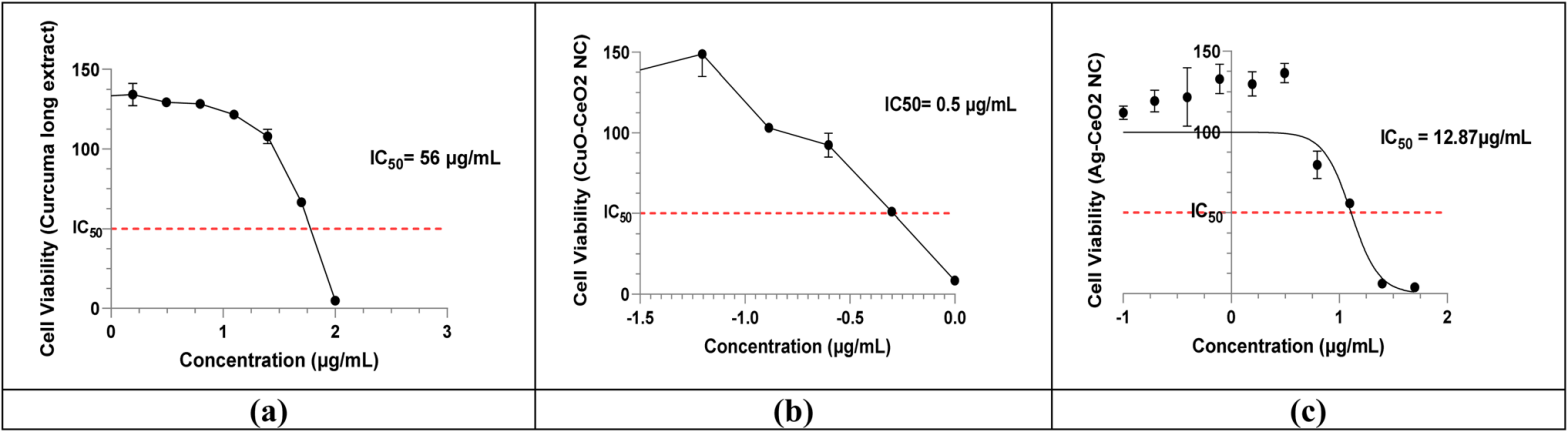
Dose–response curves illustrate the concentration-dependent cytotoxicity of (a) *C. longa* extract, (b) CuO/CeO_2_, and (c) Ag/CeO_2_ NCs.

The cytotoxic activity of turmeric extract and nanocomposites was evaluated by MTT assays against MCF-7 breast cancer cells. The three samples exhibited concentration-dependent cytotoxicity (Fig. 10). The relatively high IC_50_ value of turmeric extract (IC_50_ = 56 µg mL^−1^) suggests that it exhibits moderate cytotoxic activity against MCF-7 cells. This could be due to the presence of bioactive compounds in the extract that may interfere with cellular processes.^89,90^ The obvious lower IC_50_ value of CuO/CeO_2_ NC (IC_50_ = 0.5071 µg mL^−1^) indicates that it exhibits significantly higher cytotoxic activity compared to the turmeric extract. The enhanced cytotoxicity might be due to the unique properties of the CuO/CeO_2_ nanomaterial, such as its ability to generate reactive oxygen species or interact with cellular components.^91,92^ The IC_50_ value of Ag/CeO_2_ NC (IC_50_ = 12.87 µg mL^−1^) is lower than that of turmeric extract but higher than CuO/CeO_2_ NC, suggesting intermediate cytotoxic activity. The presence of silver nanoparticles in this nanocomposite might contribute to its cytotoxic effects.^93^ These results suggest that CuO/CeO_2_ NC exhibits the highest cytotoxic activity, followed by Ag/CeO_2_ NC and turmeric extract.

#### 3.6.2. TEM analysis of NC-treated breast cancer cells

**Fig. 11:**
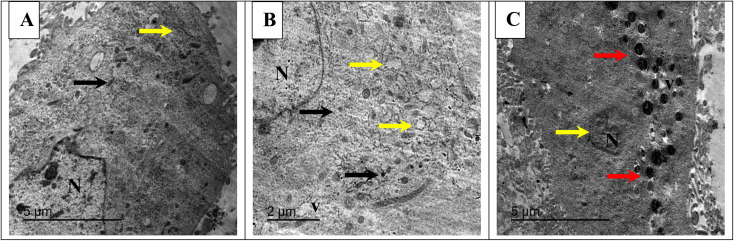
TEM of breast cancer cells (MCF-7) treated and untreated with nanocomposites. (A) Untreated control, showing a normal nucleus (N), mitochondria (black arrow), and endoplasmic reticulum (yellow arrow). (B) Cells treated with CuO/CeO_2_ NCs, showing a nearly normal nucleus (N), atrophied mitochondria (black arrow), vacuolated cytoplasm (v), and deleted endoplasmic reticulum (yellow arrow). (C) Cells treated with Ag/CeO_2_ NCs, exhibiting necrotic nuclei characterized by chromatin condensation (indicated by a yellow arrow) and vacuolated cytoplasm (v). Red arrows point to numerous lysosomes, suggesting the activation of autophagy.

TEM analysis was conducted to visualize the morphological changes induced by the treatment of MCF-7 breast cancer cells with turmeric extract, CuO/CeO_2_ NC, and Ag/CeO_2_ NC. The TEM images of untreated MCF-7 cells (Fig. 11A) revealed normal cellular structures, including a well-defined nucleus, mitochondria, and endoplasmic reticulum. This indicates that the cells were healthy and functioning normally before treatment.

The TEM images of cells treated with CuO/CeO_2_ NC showed several morphological changes (Fig. 11B). The nucleus appeared nearly normal, suggesting that DNA integrity was not severely compromised. However, the mitochondria exhibited signs of atrophy, indicating impaired energy production.^94^ Additionally, the cytoplasm was vacuolated, and the endoplasmic reticulum was disrupted, suggesting cellular damage and dysfunction.^95^ The TEM images of cells treated with Ag/CeO_2_ NC revealed even more severe morphological changes (Fig. 11C). The nucleus appeared necrotic with chromatin condensation, indicating severe cellular damage.^96^ The cytoplasm was also vacuolated, and numerous lysosomes were observed, suggesting increased autophagy activity.^97^

TEM demonstrates that both CuO/CeO_2_ NC and Ag/CeO_2_ NC exert cytotoxic effects on MCF-7 cells. CuO/CeO_2_ NC induced mitochondrial damage and cytoplasmic alterations, while Ag/CeO_2_ NC caused severe nuclear damage and autophagy. The observed morphological changes suggest that both nanocomposites may induce cell death through multiple mechanisms, including mitochondrial dysfunction, oxidative stress, and autophagy.^98,99^

MCF-7 breast cancer cells show different mechanisms of cytotoxicity against CuO/CeO_2_ and Ag/CeO_2_ nanocomposites.^100^ Cellular energy production through the mitochondria encounters damage from CuO/CeO_2_ NC, which generates ROS^101^ to cause oxidative stress. Copper-based CuO releases free copper ions that activate Fenton reactions to worsen the oxidative damage. Oxidative stress becomes stronger when CeO_2_ combines its redox-active properties with the process,^102^ which results in apoptotic or necrotic cell death. The cytotoxic mechanism of Ag/CeO_2_ NC includes severe nuclear damage leading to DNA fragmentation and chromatin condensation.^103^ Silver nanoparticles (Ag NPs) display genomic toxicity through DNA breakages that similarly damage cell proteins, causing destructive changes in nuclear structures.^104^ The combination of CeO_2_ nanoparticles seems to strengthen the cellular process of autophagy, which may result in autophagic cell death when unregulated. The joint activity between Ag and CeO_2_ produces increased cell-killing power within Ag/CeO_2_ NC. The different ways in which copper oxide (CuO) and silver (Ag) act on cellular components combined with their separate ion release methods explain their varied toxic effects on cells. CuO/CeO_2_ induces extensive oxidative stress,^105^ but Ag/CeO_2_ mainly affects DNA integrity and causes autophagic cell destruction through its actions.^106^ Some nanocomposite combinations using these metal oxides become more toxic than single compound elements. This discovery leads to valuable knowledge for better cancer treatment methods.^107,108^

## 4. Conclusion

This study presents a green synthesis of Ag/CeO_2_ and CuO/CeO_2_ NCs using *C. longa* extract. Characterization confirmed the formation of well-dispersed nanoparticles with spherical or slightly elongated morphology. The interaction between the extract and metal ions during bioreduction was evident from the UV-visible spectroscopy data. The nanoparticles exhibited comparable hydrodynamic sizes and slightly positive zeta potentials. The Ag/CeO_2_ and CuO/CeO_2_ NCs retained significant antioxidant activity similar to the turmeric extract according to DPPH assays, suggesting their potential for various applications. The observed antioxidant activity of the nanocomposites can be due to the incidence of phytochemicals and the synergistic effects of the metal oxides. On the other hand, this study highlights the potential of green synthesis of nanocomposites as antifungal agents and for cancer therapy. CuO/CeO_2_ and Ag/CeO_2_ NCs showed antifungal activity against pathogenic *M. phaseolina*. CuO/CeO_2_ NC was more effective than Ag/CeO_2_ NC, with MIC values of 29 µg mL^−1^ and 49 µg mL^−1^, respectively. TEM analysis verified that the nanocomposites likely interacted with the fungal cell wall, causing damage and inhibiting growth. Specifically, CuO/CeO_2_ and Ag/CeO_2_ NCs showed stronger cytotoxicity against MCF-7 breast cancer cells than turmeric extract. CuO/CeO_2_ was particularly effective, with an IC_50_ value of 0.5071 µg mL^−1^. TEM analysis revealed that these nanocomposites caused damage to the mitochondria, cytoplasm, and the nucleus, suggesting their potential to disrupt cellular functions and induce cell death. More investigation is needed to fully elucidate their action mechanisms and evaluate their potential as anticancer agents.

Future research should address these specified points when building upon the Ag/CeO_2_ and CuO/CeO_2_ NCs applications. Research should examine the antioxidant-, antifungal-, and anticancer processes operated by these nanocomposites by studying their effects at the organism and biochemical levels. Safety evaluation with therapeutic measurements needs to be conducted through *in vivo* experiments to assess these nanocomposites for clinical applications. Scientists should assess the prolonged effects of human cell exposure and ecosystem changes due to exposure to these nanomaterials. The complete commercial implementation of these nanocomposites requires studies on large-scale manufacturing processes and assessments of their environmental stability.

## Data availability

The datasets generated and/or analyzed during the current study are available in the ESI.† Additional data are available from the corresponding author upon reasonable request.

## Conflicts of interest

There is no conflict of interest to declare.

## Supplementary Material

RA-015-D5RA00739A-s001
