# Supplementary material for: Eco-friendly synthesis of Ag/CeO2 and CuO/CeO2 nanocomposites using Curcuma longa extract and assessment of their antioxidant, antifungal, and cytotoxic activities
Source: RSC Adv. 2025 Apr 17;15(16):12100–16. doi: 10.1039/d5ra00739a (PMC12005080; doi:10.1039/d5ra00739a)
Supplement: RA-015-D5RA00739A-s001 [file RA-015-D5RA00739A-s001.pdf]

Supplementary Information (SI) for RSC Advances.
This journal is © The Royal Society of Chemistry 2025

**Supplementary Data**

# Eco-Friendly Synthesis and Characterization of Ag/$CeO_2$ and CuO/$CeO_2$ Nanocomposites *via Curcuma longa* Extract: Evaluation of Antioxidant, Antifungal, and Cytotoxic Activities

## Section S1. Instruments

The characterization and analysis of the nanocomposites were carried out using a range of advanced instruments and techniques. Phytochemical analysis and antioxidant activity were assessed using a Spekol 11 spectrophotometer (Analytik Jena AG, Germany). Fourier-transform infrared (FTIR) spectroscopy was performed on a Thermo-Fisher Nicolet IS10 Spectrophotometer in the 500-4000 $cm^{-1}$ frequency range. Zeta potential and particle size measurements were obtained with a HORIBA Scientific SZ-100 instrument. The surface morphology, shape, and elemental composition of the nanocomposites were studied using scanning electron microscopy (SEM) coupled with energy-dispersive X-ray spectroscopy (EDX) on an FEI Czech SEM instrument at 25 kV. Transmission electron microscopy (TEM) analysis was conducted using a ThermoScientific Talos F200i instrument with a carbon-coated grid for high-resolution imaging. X-ray diffraction (XRD) analysis was performed on a Pan Analytical Philips instrument to determine the material type, phase, crystallographic structure, and physical properties. Additionally, the centrifugation of the nanocomposites was carried out with a Beckman Coulter Allegra X-15R Centrifuge (Beckman Coulter, USA), and sonication was performed using an Elma Schmidbauer GmbH Sonicator (Singen, Germany). For tissue culture in cytotoxicity assay, a Hemocytometer, Infinite F50 plate reader (Tecan Trading AG, Switzerland), and Graph Pad Prism 8 software were used.

## Section S2. Reagents:

Folin-Ciocalteu reagent (Fluka, Biochemical Inc., Bucharest, Romania), Gallic acid (Biomedical Inc., Orange City, FL, USA), 1,1-Diphenyl-2-picrylhydrazyl (DPPH$^{\bullet}$), aluminum chloride, sodium hydroxide, sodium nitrite, catechin, ascorbic acid, tannic acid, and DMSO were purchased from Sigma Aldrich (St. Louis, USA). Sodium Carbonate (El-Nasr Pharmaceutical Chemicals, Cairo, Egypt). Silver nitrate ($AgNO_3$: CAS Number: 7761-88-8; purity: 99.5%), and Copper sulfate ($CuSO_4$, ≥99%) were purchased from *PIOCHEM* for

laboratory chemicals, Egypt. Cerium oxide ($CeO_2$, ≥99%) was purchased from Sigma Aldrich, St. Louis, USA.

**Section S3. Phytochemical Analysis**

**1. Total phenolic contents**

Folin-Ciocalteu (F-C) assay was used for the investigated samples to quantify the phenolic contents [1, 2]. A volume of 100 μL of the sample was pipetted into a cuvette and mixed with 5 mL of the diluted Folin-Ciocalteu reagent (1:10). Sodium carbonate solution (4 mL, 7.5%) was added and mixed again. The final volume was adjusted to 10 mL by distilled water. The mixture was incubated in the dark to stand for 30 min at 40 $^{o}$C. The absorbance of the blue-colored solution was recorded at 765 nm using a spectrophotometer. A standard curve of gallic acid (concentration range 0-100 mg/L) was prepared and the corresponding concentration versus absorbance values were plotted, a linear regression equation being calculated. The samples' absorbance values were then interpolated in the standard curve equation to estimate their phenolic content and expressed as milligrams of gallic acid equivalents (mg GAE/gr DW) Values are expressed as milligrams Gallic acid equivalents/grams of the dried sample placed on the standard curve of Gallic acid. The results were expressed as milligrams of Gallic acid equivalents/grams of the dried sample. The process involved the use of a Gallic acid standard curve ($y = 0.0062x$, $R^2 = 0.987$).

**2. Total flavonoid contents**

Aluminum chloride colorimetric assay was used to investigate the flavonoid contents of the tested samples following the procedure reported earlier [3]. In brief, the sample (100 μl) was moved to a cuvette and subsequently mixed with 4 mL of distilled water and then 0.3 mL of sodium nitrite solution (5%). The mixture was combined and allowed to stand for 5 minutes. 0.3 mL of aluminum chloride solution (10%) in ethanol was then added and mixed properly, followed by incubation for a further 5 minutes. The suspension was then mixed with 2 mL of 1 M sodium hydroxide solution thoroughly and incubated for 15 min at ambient temperature. The volume (< 10 mL) was then adjusted up to 10 mL with distilled water. The orange solution was then measured at a wavelength of 510 nm in the spectrophotometer. For the determination of flavonoid content, a standard curve was made in a concentration range of 0-100 mg/L using catechin on which absorbance values for standards and different samples were mapped against corresponding concentrations by the linear regression equation. The total content of flavonoids in the samples was determined by applying absorbance values of the samples into a standard

curve equation and expressed as milligrams of catechin equivalents (mg QE)/g DW. The total flavonoids were estimated from the following standard curve (y = 0.0028 x, $R^2$= 0.988).

**Section S4. Antioxidant activity using DPPH assay**

The antioxidant capacity of the tested samples was investigated following the DPPH$^{\bullet}$ colorimetric method using ascorbic acid as a standard [4]. The serial dilution of each sample was prepared by mixing the sample with methanol in an equivalent amount. DPPH$^{\bullet}$ solution was prepared in a concentration of 0.135 mM and mixed with each sample in the serial dilution with an equivalent volume. After the addition of DPPH$^{\bullet}$ solution, the samples were kept in the dark for 30 minutes at room temperature. The absorbance of each sample was measured at 517 nm in the next step. The % DPPH$^{\bullet}$ remaining was calculated by stratifying the subsequent equation (Eq. 1):

$$\% DPPH\bullet\ remaining = [DPPH\bullet]T / [DPPH\bullet]T = 0 \times 100 \quad \text{Eq. (1)}$$

The values of % DPPH$^{\bullet}$ remaining were plotted versus the sample concentration in mg /mL using an exponential curve to identify the effective concentration "$IC_{50}$". $IC_{50}$ indicated the constituted amount of antioxidants needed to decrease the initial concentration of DPPH$^{\bullet}$ solution by 50%. The values of $IC_{50}$ point out the inverse relationship with the antioxidant capacity of the tested sample [5].

**Section S5. Statistical procedure, and software**

The results were presented as the average of three biological replicates. The results were articulated as means ± standard deviation (SD). All analyses of *Curcuma longa* extract and the nanocomposites were implemented in triplicate. The mean data of different samples were analyzed by the Statistical Package for Social Sciences (SPSS, version 21). Differences were deliberated statistically significant when *the p*-value was ≤0.05.

## Section S6. Characterization of Nanocomposites

### 1. FTIR and UV-Visible Spectroscopy

Table S1. FTIR spectra of *C. longa* extract, $Ag/CeO_2$ NC, and $CuO/CeO_2$ NC.

| Wavenumber ($cm^{-1}$) | Tentative Functional Group | *Curcuma longa* Extract | Band Shift ($Ag/CeO_2$) | Band Shift ($CuO/CeO_2$) | Interpretation |
|---|---|---|---|---|---|
| **3367** | O-H (hydroxyl) | 3367 | +29 | - | Likely from alcohols, phenols, or water in the extract. No significant shift in $CuO-CeO_2$ NC suggests a similar hydrogen bonding environment. |
| **3013, 2965, 2926, 2925, 2854** | C-H (aliphatic chains) | 3013, 2965, 2926, 2925, 2854 | -44, -40, -3 | | The minimal shift in $CuO-CeO_2$ NC indicates the presence of similar aliphatic chains. |
| **1741** | C=O (carbonyl) | 1741 | -2 | -1 | Likely from curcumin or other carbonyl-containing compounds in the extract. The minimal shift suggests a similar carbonyl environment in both NCs. |
| **1681, 1626, 1592, 1572** | Aromatic rings, conjugated C=C | 1681, 1626, 1592, 1572 | -1, | -1, | Characteristic peaks of aromatic rings potentially from curcumin or other aromatic compounds. The minimal shift suggests a similar aromatic structure in both NCs. |
| **1512** | Aromatic amines/N-heterocyclic rings (C-N) | 1512 | -1 | -1 | May arise from curcumin or other nitrogen-containing components. Minimal shift suggests similar N-containing groups in both NCs. |
| **1455-1131** | C-O (various) | 1455-1131 | - | - | Various C-O stretching vibrations from functional groups in curcumin and other components. No significant shift suggests a similar C-O environment in both NCs. |
| **1033** | Fingerprint region (complex) | 1033 | | | Complex region with overlapping peaks. May contain additional functional group information. |
| **1033** | Ce-O (cerium oxide) | - | 1033 | 1033 | Characteristic peak confirming the presence of $CeO_2$ in both NCs. |
| **~3000 (broad)** | O-H (hydroxyl, associated) | - | - | | A possible weak broad peak is obscured by stronger peaks in this region. This may indicate the presence of surface hydroxyls on NCs. |
| **~2900-2800** | C-H (organic moieties) | - | Presence observed (2969, 2925, 2857) | Presence observed (3013, 2966, 2925, 2854) | Organic moieties are likely from residual surfactants or adsorbed hydrocarbons during synthesis. $CuO-CeO_2$ NC might have slightly less contamination compared to $Ag-CeO_2$ NC. |
| **1319, 1280** | C-O (carbonate/organic) | 1319, 1280 | -18, -57 | -11, -3 | Possible contribution from carbonates or residual organic compounds. $CuO-CeO_2$ NC might have slightly less contamination compared to $Ag-CeO_2$ NC. |

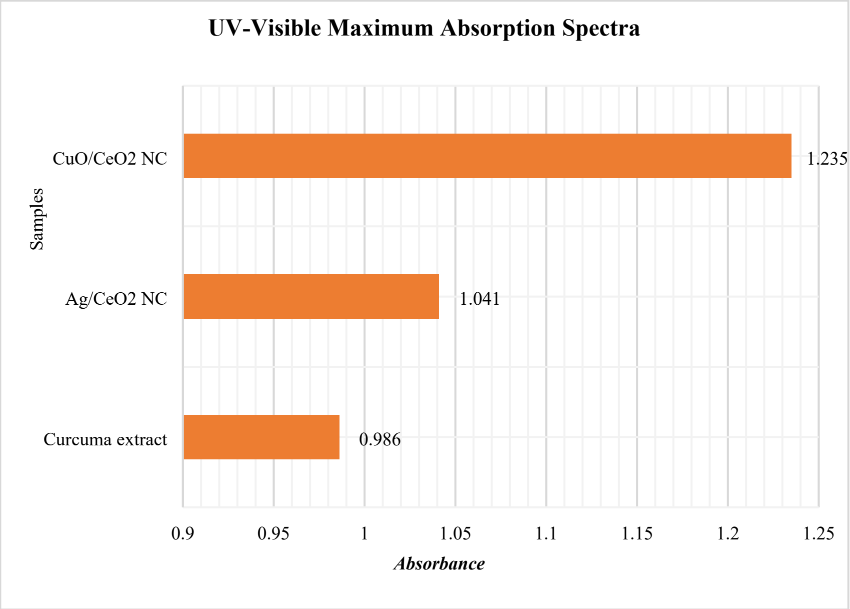


**Figure S1**. A comparison of the maximum absorption peaks of the varied samples recorded by UV-visible spectroscopy.

## 2. Zeta potential and DLS

Table S2. Characterization of $Ag/CeO_2$ and $CuO/CeO_2$ nanoparticles, including zeta potential, zeta deviation, conductivity, hydrodynamic size, and polydispersity index (PDI).

| Nanoparticle | Zeta Potential (mV) | Zeta Deviation (mV) | Conductivity (mS/cm) | Zeta Average (d.nm) | PDI |
|---|---|---|---|---|---|
| **$Ag/CeO_2$ NC** | 11.8 | 30.3 | 2.05 | 622.9 | 0.073 |
| **$CuO/CeO_2$ NC** | 9.01 | 17.7 | 1.83 | 602.9 | 0.027 |

## 3. Energy-Dispersive X-ray Spectroscopy (EDX)

Table S3. Energy-Dispersive X-ray Spectroscopy (EDX) of $Ag/CeO_2$ and $CuO/CeO_2$ nanoparticles.

| Element | Weight % ($Ag/CeO_2$) | Atomic % ($Ag/CeO_2$) | Weight % ($CuO/CeO_2$) | Atomic % ($CuO/CeO_2$) |
|---|---|---|---|---|
| **O** | 14.58 | 57.47 | 19.32 | 59.85 |
| **Zn** | 2.78 | 2.68 | 4.39 | 3.33 |
| **Ag** | 19.61 | 11.47 | - | - |
| **Ce** | 63.04 | 28.38 | 54.14 | 19.15 |
| **Cu** | - | - | 21.33 | 16.64 |

## 4. XRD Analyses

**Anchor Scan Parameters of Ag/$CeO_2$ NC**

Scan Axis: Gonio
Start Position [°2Th.]: 5.0100
End Position [°2Th.]: 89.9900
Step Size [°2Th.]: 0.0200
Scan Step Time [s]: 0.9500
Scan Type: Continuous
Offset [°2Th.]: 0.0000
Divergence Slit Type: Fixed
Divergence Slit Size [°]: 0.4785
Specimen Length [mm]: 10.00
Receiving Slit Size [mm]: 0.1000
Measurement Temperature [°C]: 25.00
Anode Material: Cu
K-Alpha1 [Å]: 1.54060
K-Alpha2 [Å]: 1.54443
K-Beta [Å]: 1.39225
K-A2 / K-A1 Ratio: 0.50000
Generator Settings: 30 mA, 40 kV
Diffractometer Type: 0000000000005545
Diffractometer Number: 0
Goniometer Radius [mm]: 240.00
Dist. Focus-Diverg. Slit [mm]: 91.00
Incident Beam Monochromator: No
Spinning: No

Table S4. Peak List of Ag/$CeO_2$ NC.

| Pos.[°2Th.] | Height[cts] | FWHM[°2Th.] | d-spacing[Å] | Rel.Int.[%] | Tipwidth[°2Th.] |
|---|---|---|---|---|---|
| 28.3382 | 203.24 | 0.1574 | 3.14945 | 100.00 | 0.1889 |
| 32.9168 | 61.93 | 0.1574 | 2.72109 | 30.47 | 0.1889 |
| 37.9536 | 12.89 | 0.2362 | 2.37076 | 6.34 | 0.2834 |
| 47.2637 | 117.24 | 0.2755 | 1.92322 | 57.69 | 0.3306 |
| 56.1385 | 90.17 | 0.1968 | 1.63841 | 44.37 | 0.2362 |
| 58.8921 | 16.08 | 0.3149 | 1.56821 | 7.91 | 0.3779 |
| 64.3399 | 2.08 | 0.9446 | 1.44795 | 1.02 | 1.1336 |
| 69.3103 | 14.31 | 0.3936 | 1.35577 | 7.04 | 0.4723 |
| 76.5791 | 29.46 | 0.3936 | 1.24418 | 14.49 | 0.4723 |
| 78.9543 | 21.00 | 0.3936 | 1.21260 | 10.33 | 0.4723 |
| 88.2705 | 28.89 | 0.5760 | 1.10619 | 14.21 | 0.6912 |

**Anchor Scan Parameters of $CuO/CeO_2$ NC**

| | |
|---|---|
| Operator | cwi |
| Raw Data Origin | XRD measurement (*.XRDML) |
| Scan Axis | Gonio |
| Start Position [°2Th.] | 5.0100 |
| End Position [°2Th.] | 89.9900 |
| Step Size [°2Th.] | 0.0200 |
| Scan Step Time [s] | 0.9500 |
| Scan Type | Continuous |
| Offset [°2Th.] | 0.0000 |
| Divergence Slit Type | Fixed |
| Divergence Slit Size [°] | 0.4785 |
| Specimen Length [mm] | 10.00 |
| Receiving Slit Size [mm] | 0.1000 |
| Measurement Temperature [°C] | 25.00 |
| Anode Material | Cu |
| K-Alpha1 [Å] | 1.54060 |
| K-Alpha2 [Å] | 1.54443 |
| K-Beta [Å] | 1.39225 |
| K-A2 / K-A1 Ratio | 0.50000 |
| Generator Settings | 30 mA, 40 kV |
| Diffractometer Type | 0000000000005545 |
| Diffractometer Number | 0 |
| Goniometer Radius [mm] | 240.00 |
| Dist. Focus-Diverg. Slit [mm] | 91.00 |
| Incident Beam Monochromator | No |
| Spinning | No |

Table S5. Peak List of $CuO/CeO_2$ NC.

| Pos. [°2Th.] | Height [cts] | FWHM [°2Th.] | d-spacing [Å] | Rel. Int. [%] | Tip width [°2Th.] |
|---|---|---|---|---|---|
| 17.1654 | 41.68 | 0.1574 | 5.16585 | 8.94 | 0.1889 |
| 18.0716 | 30.37 | 0.1181 | 4.90882 | 6.51 | 0.1417 |
| 19.9105 | 64.98 | 0.1181 | 4.45940 | 13.94 | 0.1417 |
| 22.1521 | 17.52 | 0.2362 | 4.01296 | 3.76 | 0.2834 |
| 24.2465 | 32.48 | 0.1771 | 3.67086 | 6.96 | 0.2125 |
| 25.8691 | 45.20 | 0.1181 | 3.44418 | 9.69 | 0.1417 |
| 27.2117 | 50.42 | 0.1574 | 3.27721 | 10.81 | 0.1889 |
| 28.3921 | 466.33 | 0.1771 | 3.14360 | 100.00 | 0.2125 |
| 29.4980 | 24.93 | 0.1574 | 3.02821 | 5.35 | 0.1889 |
| 31.5037 | 30.90 | 0.1574 | 2.83984 | 6.63 | 0.1889 |
| 32.9705 | 135.68 | 0.1771 | 2.71679 | 29.09 | 0.2125 |
| 35.7204 | 34.29 | 0.1181 | 2.51369 | 7.35 | 0.1417 |
| 39.3342 | 23.68 | 0.1968 | 2.29067 | 5.08 | 0.2362 |
| 47.3354 | 199.94 | 0.2362 | 1.92047 | 42.87 | 0.2834 |
| 56.2019 | 153.78 | 0.1181 | 1.63672 | 32.98 | 0.1417 |
| 59.0040 | 22.78 | 0.3542 | 1.56550 | 4.88 | 0.4251 |
| 69.3261 | 24.94 | 0.3149 | 1.35550 | 5.35 | 0.3779 |
| 76.5873 | 43.20 | 0.3936 | 1.24406 | 9.26 | 0.4723 |
| 78.9569 | 28.78 | 0.2362 | 1.21257 | 6.17 | 0.2834 |
| 88.3031 | 29.94 | 0.4800 | 1.10586 | 6.42 | 0.5760 |

**Section S7. Results of the phytochemical profile, antioxidant, and anticancer activities.**

Table S6. Phytochemical results of the investigated sample.

| Sample | Phenolic Contents (mg GAE/g) | Flavonoid Contents (mg CE/g) |
|---|---|---|
| **Turmeric extract** | 215.3 ± 16.40 | 154.5 ± 19.10 |
| **Ag/$CeO_2$ NC** | 148.3 ± 7.77 | 92.89 ± 13.40 |
| **CuO/$CeO_2$ NC** | 174.1 ± 7.46 | 91.64 ± 8.46 |

**GAE:** Gallic acid equivalents. **CE:** Catechin equivalents. All values in the table are expressed as mean ± standard deviation. The experiments were run for triplicates.

Table S7. The results of the antioxidant activity of the tested samples using DPPH assay.

| Sample | Concentrations (mg/mL) | % Remaining DPPH (mean ± SD) | % Scavenging Activity (mean ± SD) | $IC_{50}$ (mg/mL) (mean ± SD) |
|---|---|---|---|---|
| **Turmeric extract** | 0.053 | 42.64 ± 1.58 | 56.99 ± 1.68 | 0.042 ± 0.004 |
| | 0.026 | 62.90 ± 0.80 | 37.20 ± 1.20 | |
| | 0.013 | 73.46 ± 2.44 | 26.21 ± 2.96 | |
| | 0.007 | 88.98 ± 3.50 | 9.69 ± 2.79 | |
| **Ag/$CeO_2$ NC** | 0.100 | 73.96 ± 1.16 | 25.67 ± 1.40 | 0.242 ± 0.020 |
| | 0.050 | 80.25 ± 0.95 | 19.41 ± 1.04 | |
| | 0.025 | 88.02 ± 1.39 | 11.95 ± 1.45 | |
| | 0.013 | 93.64 ± 0.71 | 5.02 ± 1.42 | |
| **CuO/$CeO_2$ NC** | 0.117 | 37.23 ± 1.24 | 63.10 ± 1.27 | 0.081 ± 0.004 |
| | 0.058 | 61.24 ± 2.66 | 38.76 ± 2.15 | |
| | 0.029 | 75.52 ± 1.23 | 24.44 ± 1.93 | |
| | 0.015 | 85.59 ± 1.45 | 14.41 ± 1.87 | |
| **Ascorbic acid** | 0.062 | 15.27 ± 0.48 | 84.73± 1.58 | 0.022± 0.176 |
| | 0.031 | 39.08± 0.19 | 60.92± 2.34 | |
| | 0.016 | 61.07± 0.73 | 38.93± 1.82 | |
| | 0.008 | 74.81± 1.16 | 25.19± 1.07 | |

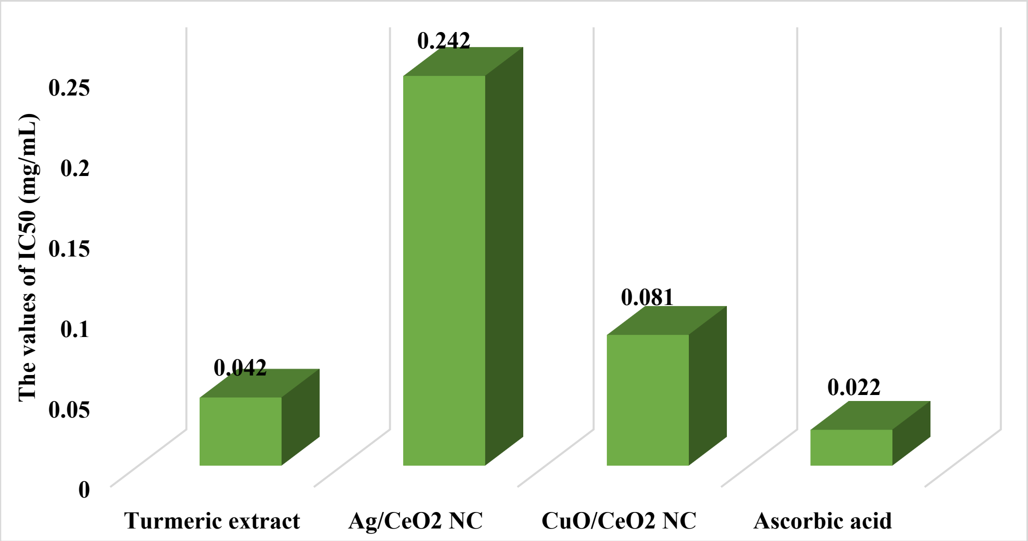


**Figure S2. A comparison of the $IC_{50}$ (mg/mL) values calculated for the test samples.**

Table S8. The FTIR spectra of the treated and untreated *Macrophomina phaseolina* with $Ag/CeO_2$ and $CuO/CeO_2$ NCs.

| Wavenumber ($cm^{-1}$) | Tentative Functional Group | Untreated | Band Shift ($Ag/CeO_2$) | Band Shift ($CuO/CeO_2$) | Interpretation |
|---|---|---|---|---|---|
| **3274** | O-H stretching (carbohydrates, proteins, water) | - | +83 $cm^{-1}$ | +6 $cm^{-1}$ | Possible alteration in hydrogen bonding or changes in water content. |
| **2923** | C-H stretching (aliphatic chains) | - | - | - | No significant change in aliphatic chain content. |
| **2852** | C-H stretching (aliphatic chains) | - | - | - | No significant change in aliphatic chain content. |
| **2264** | C≡N stretching (alkynes/nitriles) | - | Not Present | Not Present | Absence suggests no presence of these functional groups. |
| **2116** | C=C stretching (alkenes) | - | Not Present | Not Present | Absence suggests no presence of these functional groups. |
| **1834** | C=O stretching (aldehydes, ketones) | - | - | - | No significant change in carbonyl groups. |
| **1795** | C=O stretching (carboxylic acids) | - | Not Present | Not Present | Absence suggests no significant change in carboxylic acids. |
| **1699** | C=O stretching (conjugated ketones) | - | Not Present | Not Present | Absence suggests no significant change in conjugated ketones. |
| **1648** | Amide I (proteins) | - | -1 $cm^{-1}$ | -2 $cm^{-1}$ | A slight shift might indicate minor changes in the protein's secondary structure. |
| **1616** | Amide II (proteins) | - | -1 $cm^{-1}$ | -2 $cm^{-1}$ | Similar to Amide I, suggests minor protein structural changes. |
| **1558** | N-H bending (amides) | - | -13 $cm^{-1}$ | - | Potential decrease in amide content or alteration in hydrogen bonding. |
| **1509** | Aromatic C=C stretching (aromatic rings) | - | Not Present | Not Present | Absence suggests no presence of aromatic compounds. |
| **1459** | C-H bending (methylene/methyl groups) | - | +2 $cm^{-1}$ | -1 $cm^{-1}$ | Minor shift might indicate changes in $CH_2$ or $CH_3$ groups. |
| **1422** | O-H deformation (carboxylic acids) | - | - | - | No significant change in carboxylic acid O-H bending. |

| | | | | | |
|---|---|---|---|---|---|
| **1369** | C-H bending (methylene/methyl groups) | - | -1 $cm^{-1}$ | - | Minor shift might indicate changes in $CH_2$ or $CH_3$ groups. |
| **1273** | Ester C-O stretching (esters) | - | Not Present | Not Present | Absence suggests no presence of ester functional groups. |
| **1241** | P=O stretching (phosphates) | Not Present | +42 $cm^{-1}$ | Not Present | Appearance suggests the introduction of phosphates, possibly from cellular components. |
| **1181** | C-O stretching (alcohols, ethers) | Not Present | +24 $cm^{-1}$ | Not Present | Appearance suggests the introduction of C-O bonds from alcohols or ethers. |
| **1159** | C-O stretching (carbohydrates) | - | -7 $cm^{-1}$ | -4 $cm^{-1}$ | The shift suggests a potential alteration in carbohydrate structure. |
| **1072** | C-O stretching (carbohydrates) | - | +2 $cm^{-1}$ | - | The minor shift suggests possible changes in carbohydrate structure. |
| **1032** | C-O stretching (carbohydrates) | - | +3 $cm^{-1}$ | -3 $cm^{-1}$ | Inconsistent shift; needs further investigation for carbohydrate changes. |

**Figure S3** shows the growth patterns of *Macrophomina phaseolina* untreated and treated with nanocomposites. The untreated *M. phaseolina* produces a typical fungal colony that spreads outward from its middle in a pattern resembling a network through its hyphal extensions. *M. phaseolina* demonstrates such growth patterns because of its natural pathogen behavior that serves as a baseline evaluation for treatment outcome assessment. The expansive fungal growth serves as evidence that no antimicrobial intervention has occurred, hence demanding a need for antifungal intervention. Previous research demonstrated that *M. phaseolina* produces colonies that match the current observational findings in a standard laboratory environment. The fungal growth experienced a substantial reduction because of Ag/$CeO_2$ nanocomposites, which established an apparent inhibition zone at the nanocomposites application point. The Ag/$CeO_2$ nanocomposites succeeded in preventing *M. phaseolina* growth because silver (Ag) nanoparticles and cerium oxide ($CeO_2$) together create reactive oxygen species (ROS) and support redox reactions, thus displaying antimicrobial properties. Ag/$CeO_2$ nanocomposites show promise as an effective plant protection alternative through their use as an eco-friendly antifungal agent, according to test results. The antifungal activity of treated areas became evident because CuO/$CeO_2$ nanocomposites manifested a distinct inhibition zone. The CuO nanoparticles operably combined with $CeO_2$ particles function as strong antifungal agents by producing ROS to damage fungal cells and block growth progression. The inhibition zone from this treatment research reveals that CuO/$CeO_2$ nanocomposites should play a role as a potent ecological antifungal agent for *M. phaseolina* plant protection.

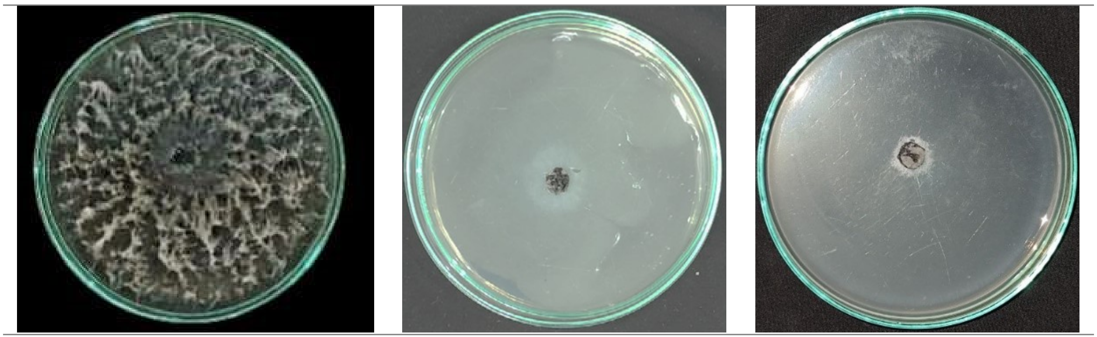

| **(a) Untreated *M. phaseolina*** | **(b) *M. phaseolina* treated with Ag/$CeO_2$ NC** | **(c) *M. phaseolina* treated with CuO/$CeO_2$ NC** |
|---|---|---|

**Figure S3**. Growth of the treated (Ag/$CeO_2$ and CuO/$CeO_2$ NCs) and untreated *M. phaseolina* on agar plates.

## Cytotoxic Activity

### Cell line and cell culture

The MCF-7 human breast cancer cell line was obtained from NAWAH Company. These cells were maintained in tissue culture flasks using Dulbecco's Modified Eagle Medium (DMEM) supplemented with 10% fetal bovine serum and 1% penicillin/streptomycin. Cells were maintained in a humidified incubator at 37 $^{o}$C with 5% $CO_2$. The culture medium was replaced every three days until the cells reached 70-80% confluence. Subsequently, the cells were trypsinized using 0.25% trypsin/EDTA and counted. To count the cells, the trypsinized cells were resuspended in an equal volume of fresh media. A 10 µL aliquot of the cell suspension was transferred to a 1.5 mL microcentrifuge tube and mixed with 10 µL of 0.4% trypan blue solution. After cleaning and moistening the coverslip of a hemocytometer, both sides of the chamber were filled with 10 µL of the cell suspension. The viable cells were counted under a 10x magnification microscope. The concentration of viable cells was calculated using Eq. (2).

$$Average\ of\ viable\ cell = \frac{Total\ no.\ of\ viable\ cells}{Total\ no.\ of\ squares\ \times\ dilution\ factor} \times 10^4 \qquad \text{Eq. (2)}$$

### MTT assay

Cells were seeded at a density of 5 × $10^4$ cells/mL (100 µL/well) in a 96-well plate. After overnight incubation at 37°C and 5% $CO_2$, the cells were treated with various concentrations of the tested samples (50, 25, 12.5, 6.25, 3.125, and 1.25 µM). DMSO (0.5% V/V) served as a negative control. Following a 48-hour incubation at 5% $CO_2$ and 37°C, 100 µL of MTT solution (5 mg/mL PBS) was added to each well and incubated for 4 hours [6]. Viable cells reduced MTT to formazan crystals. Subsequently, 100 µL of DMSO was added to dissolve the formazan crystals, and the mixture was incubated for 15 minutes at 37°C and 5% $CO_2$. The absorbance of each well was measured at $\lambda$= 570-630 nm using an Infinite F50 plate reader. This 8-channel absorbance reader measures the optical density (OD) of liquids in 96-well microplates. The LED light source emits light that passes through an optical filter to select the desired wavelength for absorbance measurement. The filtered light beam is split into eight optical fibers that focus on eight wells of the microplate. The light beams that pass through the wells are focused by optical lenses and detected by photodiodes. Therefore, the Infinite F50 plate reader can measure eight wells simultaneously, corresponding to one column of a 96-well microplate [7]. Each assay was repeated at least three times, and cell viability was calculated using Eq. (3). The $IC_{50}$ values of the drugs were determined using GraphPad Prism 8 software.

$$Viability = \frac{(Sample_{OD} - blank_{OD})}{(control_{OD} - blank_{OD})} * 100\% \qquad \text{Eq. (3)}$$

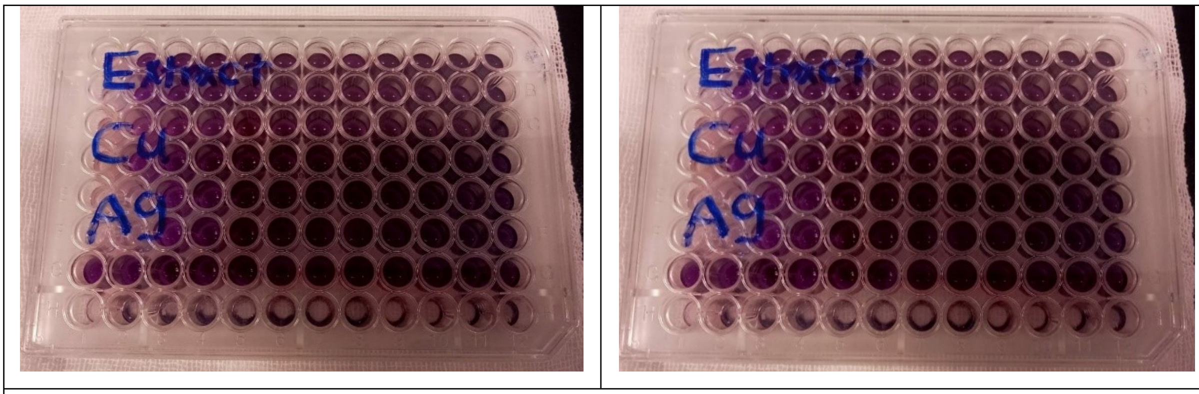


(a)

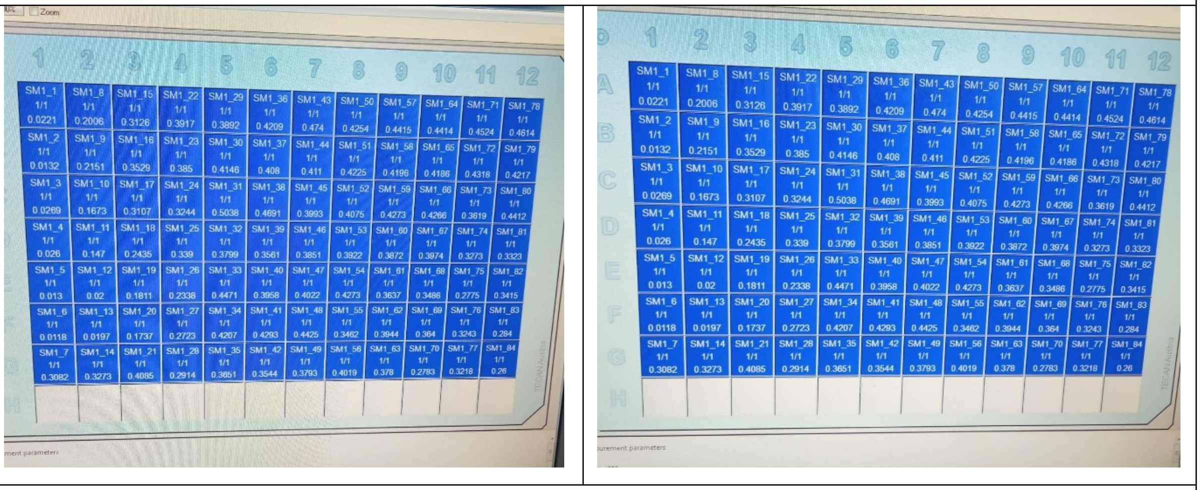


(b)

**Figure S4**. (a) A 96-well plate containing samples labeled "Extract," "Cu," and "Ag." The wells are filled with a purple solution, likely indicating a positive result in an MTT assay. (b) The reads of cytotoxicity assay, with values representing absorbance measurements. The labels referred to the different concentrations of the tested nanocomposites. The plate reader used is a Tecan Infinite F50, and the serial number is 1311009074.

## References


1. C. Cupp-Enyard, *JoVE (Journal of Visualized Experiments)*, 2008, DOI: https://dx.doi.org/10.3791/899, e899.
2. J. C. Sánchez-Rangel, J. Benavides, J. B. Heredia, L. Cisneros-Zevallos and D. A. Jacobo-Velázquez, *Analytical methods*, 2013, **5**, 5990-5999.
3. J. Zhishen, T. Mengcheng and W. Jianming, *Food chemistry*, 1999, **64**, 555-559.
4. D. D. Kitts, A. N. Wijewickreme and C. Hu, *Molecular and cellular biochemistry*, 2000, **203**, 1-10.
5. I. Parejo, C. Codina, C. Petrakis and P. Kefalas, *Journal of Pharmacological and toxicological Methods*, 2000, **44**, 507-512.
6. P. W. Sylvester, *Drug Design and Discovery: Methods and Protocols*, 2011, 157-168.
7. H. Liu, S. Iketani, A. Zask, N. Khanizeman, E. Bednarova, F. Forouhar, B. Fowler, S. J. Hong, H. Mohri and M. S. Nair, *Nature communications*, 2022, **13**, 1891.
